# Wind-Tunnel Simulation of Weakly and Moderately Stable Atmospheric Boundary Layers

**DOI:** 10.1007/s10546-018-0337-7

**Published:** 2018-02-21

**Authors:** Philip E. Hancock, Paul Hayden

**Affiliations:** EnFlo Laboratory, Department of Mechanical Engineering Sciences, University of Surrey, Guildford, Surrey GU2 7XH UK

**Keywords:** Atmospheric boundary layer, Local similarity, Stable stratification, Wind-tunnel simulation

## Abstract

The simulation of horizontally homogeneous boundary layers that have characteristics of weakly and moderately stable atmospheric flow is investigated, where the well-established wind engineering practice of using ‘flow generators’ to provide a deep boundary layer is employed. Primary attention is given to the flow above the surface layer, in the absence of an overlying inversion, as assessed from first- and second-order moments of velocity and temperature. A uniform inlet temperature profile ahead of a deep layer, allowing initially neutral flow, results in the upper part of the boundary layer remaining neutral. A non-uniform inlet temperature profile is required but needs careful specification if odd characteristics are to be avoided, attributed to long-lasting effects inherent of stability, and to a reduced level of turbulent mixing. The first part of the wind-tunnel floor must not be cooled if turbulence quantities are to vary smoothly with height. Closely horizontally homogeneous flow is demonstrated, where profiles are comparable or closely comparable with atmospheric data in terms of local similarity and functions of normalized height. The ratio of boundary-layer height to surface Obukhov length, and the surface heat flux, are functions of the bulk Richardson number, independent of horizontal homogeneity. Surface heat flux rises to a maximum and then decreases.

## Introduction

With the cessation of daytime convective motions, or the absence of such motions over a persistently cold surface, a stable atmospheric boundary layer is a naturally occurring phenomenon. Practical interest in the nature and behaviour of stable boundary layers is wide-ranging from, for example, the large scales inherent in numerical weather prediction and climate-change modelling to the small scales of modelling dispersion of pollutants in urban environments, or the modelling of turbine wakes in a wind farm. Measurements in the atmosphere, compared with laboratory-based measurements, are difficult and expensive to make, and are subject to boundary conditions that may be complex or unknown. Laboratory measurements on the other hand provide well-defined boundary conditions, but are limited to what can be represented. For instance, it is not possible to represent Coriolis forces in a wind tunnel, or the high Reynolds number of the atmosphere, thereby limiting the range between the largest and smallest scales of motion. (Limitations also occur for other methods, such as large-eddy or direct-numerical simulation, of course.) There is, nevertheless, a clear place for the study of stable boundary layers using wind-tunnel simulation techniques.

The simplest description of the atmospheric boundary layer (ABL) is one in which, while there is necessarily a prevailing wind, the flow is horizontally homogeneous with quantities varying only with height. The flow evolves in time, but without horizontal variation, free, in a practical realization, of significant upstream influence. Horizontal homogeneity has been the basis of many field and theoretical studies. Although there is not an exact transformation between a temporarily-evolving flow in an idealized atmosphere and a spatially-evolving (temporarily steady) flow in a wind tunnel it is assumed the two are equivalent on the basis of local scaling.

Given the advantages of laboratory experiments, and their importance in investigating stable and convective boundary layers, there is an intrinsically interesting question around just what can be simulated in a suitably designed wind tunnel. It has long been the practice in wind engineering to use a system of tall flow generators in order to create profiles of mean velocity and turbulence quantities that have characteristics of the boundary layer, at a scale sufficiently large, in order either to avoid excessive Reynolds number dependence (for a ‘low’ freestream velocity) or to meet practical experimental requirements, or both. Here, ‘tall’ is used to mean a height comparable to the height of the boundary layer that is generated downstream. Early work was undertaken by Armitt and Counihan (1968) and Counihan (1969, 1970, 1973), and the initial reason for using flow generators was to provide a thick boundary layer without the need for a long working section, which had been a strategy adopted by others at that time (see Armitt and Counihan 1968), though without some form of flow generator this would still not have provided characteristics typical of the ABL. Hohman et al. (2015) investigated refinements of the Counihan method, in comparison with the atmospheric data of ESDU (Engineering Sciences Data Unit 1972, 1974), though with still quite significant differences, while the earlier work of Robins (1979) shows good agreement with ESDU data. A thick boundary layer (say, about 1 m deep) is generally advantageous in terms of instrumentation and the airflow model in question, though the precise scale at which any experiment is to be conducted should be part of the design of the experiment. While the Counihan generator is an elliptically-shaped tapered wedge combined with a barrier wall, other types of generator have been employed. A more practical one involves a flat, triangular spire, which is easier to manufacture, and which gives profiles close to those based on the ESDU data (e.g. Hancock and Pascheke 2014). Details are given in Irwin (1981).

Compared with the number of wind-tunnel experiments in simulated neutral flow that in non-neutral flow is very small, e.g., Arya and Plate (1969), Ogawa et al. (1981), Meroney and Melbourne (1992), Fedorovich et al. 1996, Ohya et al. (1996, 1997, 2008), Ohya (2001), Robins et al. (2001), Ohya and Uchida (2003), Chamorro and Porté-Agel (2010), Williams et al. (2017) and Van Buren et al. (2017). In terms of boundary-layer development, most have employed a fence followed by surface roughness, where the fence height is much less than that of the developed boundary-layer height. Robins et al. (2001), though, employed the Counihan system, while Hancock et al. (2013), Hancock and Pascheke (2014), Hancock and Farr (2014), Hancock and Hayden (2016) and Marucci et al. (2016) employed triangular ‘Irwin-type’ flow generators. Both Williams et al. (2017) and Van Buren et al. (2017) employed a transition trip, but for a boundary layer developed on a wind-tunnel roof, and with surface heating to provide the thermal stability.

Ideally, it would be possible to start with a set of required profiles of mean velocity, mean temperature and other quantities and then be able to determine by direct means the details of the flow generators. In practice, in the EnFlo Laboratory, the details of the flow generators for neutral flow have been established by experiment and trial and error. In this study the starting point for the simulation of a non-neutral flow has been to use generators that give a satisfactory set of profiles of mean velocity and Reynolds stresses in neutral flow. This was the approach adopted by Hancock and Pascheke (2014) for a stable layer and Hancock et al. (2013) for a convective layer, where the neutral case was closely matched to neutral atmospheric data of ESDU (2001, 2002). It is supposed that with sufficient experience it will be possible to specify flow generators that give more closely the profiles required for non-neutral flow, amounting to a generalization of the ‘Counihan technique’. Indeed, Armitt and Counihan (1968) comment that “Simulation of the neutral atmosphere is an essential step towards the complete simulation of stable and unstable atmospheres ... “. With some lapse of time, our study contributes to that aspiration.

In the experiments of Robins et al. (2001), the temperature of the inlet flow was uniform with height, while for Hancock and Pascheke (2014) the temperature at the inlet increased linearly with height, imposing an inversion above the boundary layer (which at full scale is about $0.01\,\hbox {K\,m}^{-1})$. However, the spires reached the full height of the working section, giving excessive turbulence and a velocity gradient above the top of the boundary layer, as defined by the Reynolds shear stress. For the unstable case, Hancock et al. (2013) were able to adopt an iterative strategy in which an initially uniform inlet temperature was changed to that of the profile measured in the latter part of the working section, convergence occurring after three cycles and giving Reynolds-stress profiles that agreed better with atmospheric data. This approach did not work in the present investigation, partly because the thermal layer did not initially extend to the full height of the momentum layer, and remained shallow with further iteration.

The primary objective is the development of a technique for generating an artificially thickened boundary layer that has characteristics typical of weakly and moderately stable, horizontally homogenous atmospheric boundary layers (across the whole depth), building on well-established wind-engineering techniques for neutral flow. While it was supposed at the outset that some form of non-uniform temperature profile would be needed, it was not known what form this should be, with no precedent to follow (other than the limited example of Hancock and Pascheke 2014). The main ‘yard sticks’ of success are the measurements of Caughey et al. (1979) and the local scaling systems of Nieuwstadt (1984) and Sorbjan (2010). Although primary attention is given to the flow above the surface layer, some results are also given for the surface layer itself. There is, it appears, no particular measure by which horizontal homogeneity is judged to have been achieved, and is dependent on the application in question. Here, we take it that profiles of mean velocity and other quantities change only slowly over the fetch of interest of, say, 10 boundary-layer heights. The particular application to which the present work is directed is that of wakes of wind turbines in offshore wind farms.

## A Scaling Consideration

Although the investigation is of the flow downstream of tall generators that induce a much deeper boundary layer, it is useful for a moment to consider a boundary layer growing from zero thickness, with a constant difference, $$\Delta \Theta $$, between the temperature of the freestream, $$\Theta _h $$, and that of the surface, $$\Theta _0 $$. Necessarily, the boundary layer starts as a neutral flow, which is straightforward to see from the gradient Richardson number, $$Ri= (g/\Theta )(\hbox {d}\Theta /\hbox {d}z)/(\hbox {d}U/\hbox {d}z)^{2}$$, or the bulk Richardson number, $$Ri_h =gh\Delta \Theta /(\Theta _0 U_h^{2})$$, where *g* is the acceleration due to gravity, $$\Theta $$ is the absolute temperature, *z* is the vertical distance, *h* is the boundary-layer height, *U* is the horizontal flow speed, and $$U_h $$ is the value of *U* at the top of the boundary layer. The stability in terms of Richardson number increases proportionately with height, and so increases with distance as the boundary layer grows. Clearly, a boundary layer grown in this way cannot provide horizontally homogeneous conditions.

Overlooking this last point for a moment, the surface heat flux, denoted here by $(\overline{w\theta } )_0$, is a function of distance *x* from the origin according to

1 $$\begin{aligned} (\overline{w\theta } )_0 =F(x, \Delta \Theta , \Theta _0,g, U_h ,u_{*}, z_0, z_{0\theta } ), \end{aligned}$$

where $$z_0 $$ and $$z_{0\theta } $$ are, respectively, aerodynamic and thermal roughness lengths, and $$u_{*} $$ is the friction velocity. Viscosity and thermal conductivity are ignored, assuming the Reynolds number is sufficiently large. Note that *h* is also a function of the variables on the right-hand side of Eq. 1. Assuming similarity, the development length *x* is no longer an explicit variable, and we can write this equation non-dimensionally as

2 $$\begin{aligned} \frac{h\;g\;(\overline{w\theta } )_0 }{\Theta _0 \;U_h^{3}}= F\left( \;\frac{g}{\Theta _0 }\;\frac{\Delta \Theta \;h}{U_h^{2}}, \frac{u_{*} }{U_h }, \frac{z_0 }{h}, \frac{z_{0\theta } }{z_0 }\right) . \end{aligned}$$

The left-hand side can be written as

3 $$\begin{aligned} \frac{\kappa \;h\;g\;(\overline{w\theta } )_0 }{\Theta _0 \;u_{*} ^{3}}\frac{u_{*}^{3}}{U_h^{3}}\frac{1}{\kappa }= \frac{h}{L_0 }\frac{u_{*}^{3}}{U_h^{3}}\frac{1}{\kappa }, \end{aligned}$$

where $L_{0}$ is the surface Obukhov length ($= \Theta _0 u_*^3 /(\kappa g(\overline{w\theta } )_0 )$, and $\kappa$ is the von Kármán constant. By a similar argument, the surface kinematic shear stress, denoted by $(\overline{uw} )_0$ ($=u_{*}^{2}$), can be written as

4 $$\begin{aligned} (\overline{uw} )_0 = P(x,\;\Delta \Theta ,\;\Theta _0,g,\;U_h,\;z_0,\;z_{0\theta } ), \end{aligned}$$

and then, with the same further assumptions, we have

5 $$\begin{aligned} \frac{u_{*}^{2}}{U_h^{2}} = P \left( \frac{g}{\Theta _0 }\;\frac{\Delta \Theta \;h}{U_h^{2}}, \frac{z_0 }{h}, \frac{z_{0\theta } }{z_0 }\right) . \end{aligned}$$

From Eqs. 2, 3 and 5 we obtain

6 $$\begin{aligned} \frac{h}{L}_0 = F \left( \;\frac{g}{\Theta _0 }\;\frac{\Delta \Theta \;h}{U_h^{2}}\;,\;\frac{z_0 }{h}, \frac{z_{0\theta } }{z_0 }\right) , \end{aligned}$$

where the von Kármán constant has been subsumed into the function *F*. As will be seen (in Sect. 4.6), this functional dependency is useful in the analysis of the measurements, where the effects of roughness appear secondary. A practical example is that if *h* is known (as it is approximately when flow generators are employed), then a given $$h/L_0 $$ can be attained with $$\Delta \Theta /U_h^{2}$$ according to Eq. 6.

From physical considerations, as $\Delta \Theta$ is increased, with all else constant, the surface heat transfer must at first increase. But, as the effects of stability increase with the consequential reduction in vertical transport (and an eventual complete cessation of turbulent transport^1^), the surface heat transfer is expected to reach a maximum and then decrease with further increase in $\Delta \Theta$; see Malhi (1995) and Mahrt et al. (1998) who show this to be so from atmospheric observations. As will be seen, the rise and subsequent decrease in surface heat transfer with increasing (bulk) Richardson number is observed here.

## Wind Tunnel and Instrumentation

The EnFlo wind tunnel is of a suck-down, open-return configuration, with a working section that is 20 m in length, 3.5 m in width and 1.5 m in height. It is specially designed to simulate the atmospheric boundary layer under neutral, stable or unstable conditions, where stable stratification is achieved by means of the heating elements across the working-section inlet combined with cooled working-section floor panels. These panels are each 1 m in (streamwise) length, supplied by a chilled-water system, and can be individually isolated, allowing panels to have zero heat flux. The first 1 m of the floor length has no cooling capability. In order that the flow is stratified stably (or unstably) to levels of practical interest, while at the same time maintaining reasonable overall power requirements, the maximum working-section flow velocity is restricted to about $3\,\hbox {m\,s}^{-1}$. The wind tunnel has a bell-mouth type entry with heaters and flow-smoothing screens and honeycombs to eliminate swirl, but it does not have a settling chamber and contraction upstream of the working section.

**Fig. 1:**
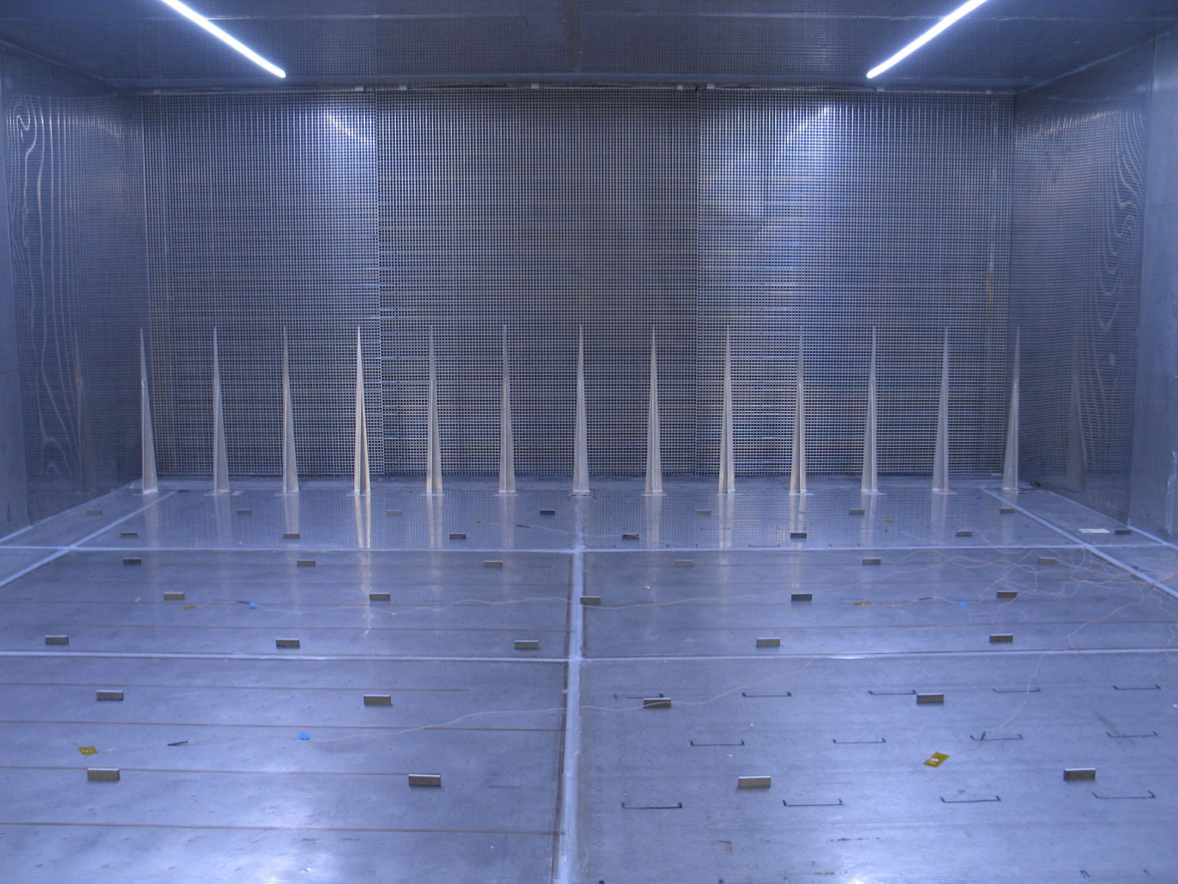
Photograph of the working-section inlet, looking upstream, showing the spires and the sparse roughness elements. The picture also shows the first 3 m of the floor, and the screen downstream of the inlet heaters

The deep boundary layer was generated by means of flat-plate spires mounted at the working-section inlet, together with sharp-edged roughness elements mounted on the floor. The thirteen spires were slightly truncated triangles with a base width of 60 mm and a tip width of 4 mm at a height of 600 mm, spaced laterally at intervals of 266 mm. They were a scaled-down version by a factor of 0.4. of the spires that had been employed previously by Hancock and Pascheke (2014). The surface roughness elements were sparsely-spaced sharp-edged blocks, as used previously but without being scaled down: 50 mm wide, 16 mm high and 5 mm thick, standing on the 50 mm $\times$ 5 mm face. They were placed in a staggered arrangement with streamwise and lateral pitches of 360 and 510 mm, respectively. With respect to the first row, the second row was displaced laterally by 255 mm, the third row by 127.5 mm, and the fourth row by 382.5 mm, to give the staggered pattern. The fifth row repeated the first, and so on. The low density of roughness elements reflects the interest in sea-surface roughness (e.g. Counihan 1975) in related work. Figure 1 shows the spires and roughness elements, and also shows the flow-smoothing screen downstream of the inlet heaters. Although the spires were geometrically the same as previously employed, it is not expected that the neutral-flow profiles match those of the earlier work. (Notwithstanding the relative difference with respect to the roughness elements, the earlier spires reached to the roof of the wind tunnel; see Irwin (1981).) Nor was it expected that the flow matches, for example, a high Reynolds number, zero-pressure gradient turbulent boundary layer, though this could be a future objective.

Measurements of mean velocity and Reynolds stresses were made using a recently calibrated Dantec FibreFlow two-component, frequency-shifted laser-Doppler anemometry (LDA) system. The probe head was held by a traversing system that hung from rails mounted beneath the wind tunnel roof. Only the streamwise and vertical velocity components were measured, where $U_{i}$ and $W_{i}$ are, respectively, the instantaneous streamwise and vertical components. *U* and *W* (no suffix) are used to denote the mean components, and *u* and *w* to denote the fluctuating components. Mean and higher-order averages were obtained using transit-time weighting. For example, the Reynolds shear stress was calculated from the equation $\overline{uw} =\sum _1^N {\omega _i (U_i } -U)(W_i -W)/\sum _1^N {\omega _i }$, where $\omega$ is the particle transit time, *N* is the number of samples, and suffix *i* denotes the $i{\mathrm{th}}$ of *N* samples.

Mean temperatures were measured using thermistor probes and the fluctuating temperature by means of a cold-wire, fast-response probe held at 4 mm behind the LDA measurement volume in order to measure the kinematic turbulent heat fluxes, where the advection time was calculated using $U_{i}$ in order to correct for the displacement. Heist and Castro (1998) give a brief review of this technique for measuring turbulent heat flux. They used an essentially identical system in the same wind tunnel in comparable conditions and showed that the frequency response of the cold wire was sufficient to include the first decade of the inertial subrange, to about 300 Hz. The separation of 4 mm is equivalent to about the same frequency at a typical mean convection velocity. As here, they also observed no significant degradation of frequency response as a result of the flow seeding. Compared with an isothermal flow, LDA measurements in principle are affected by spatial and temporal temperature variations along the length of the beams and the back-scatter light paths, demonstrated to be negligible by Hancock and Pascheke (2014). At a distance of 4 mm the blockage effect of the cold-wire probe on *U* was about 1%, for which a correction has been made, with no detectable influence on turbulence quantities. There was also a small blockage effect of the probe-traversing mechanism when the probe was above a height of about 550 mm, requiring a correction not exceeding 1%. The cold-wire probe was calibrated against a thermistor, itself used against a standard calibration; differences between thermistors were $< 0.1\,^{\circ }\hbox {C}$. Sample times were 3 min at a data rate of typically 100 Hz. Statistical errors in mean velocity were within $\pm \,0.5\%$ and within $\pm \,5\%$ for second-order momentum and thermal moments, to 95% confidence level; errors in spatial position measurements were negligible.

Lateral uniformity was assessed by means of lateral traverses at $z = 300\,\hbox {mm}$, that is, at about half the height of the artificially thickened boundary layer. The absence of a settling chamber and contraction implies that the lateral uniformity is not as high as would be expected of a high-quality conventional wind tunnel. The lateral variations for case 22 (see Table 1) are typical. Over a lateral distance of 3*h* the standard deviations, as a fraction of the respective local mean, are as follows: *U*, 0.7%; $\overline{u^{2}}$, 7%; $\overline{w^{2}}$, 5%; $-\,\overline{uw}$, 10%; $\overline{w\theta }$, 10%, $\overline{\theta ^{2}}$, 8%; $\Theta$, 0.7%, where the last is with respect to the difference $\Delta \Theta$, with no indication of lateral variations being associated with the lateral spacing of the spires. (The first measurement station was at a downwind distance of $24 \times$ the lateral spacing.)

Surface values of shear stress and heat flux were determined from linear extrapolation of the corresponding profiles, over about the inner third of the boundary layer. The viscous and thermal conduction contributions over the measured profiles did not exceed about 3.5 and 7%, respectively, with extrapolation expected to be within about $\pm \,6\%$ for both. The various quantities presented are for the most part not normalized on surface quantities; rather, for momentum quantities, either the reference or the freestream velocity is employed, as the primary attention is to the development of the flow above the surface layer. The use of a single, fixed reference velocity is particularly relevant in assessing horizontal homogeneity, and for this reason, thermal quantities are mostly left in dimensional form.

The wind-tunnel reference velocity, $U_{{Ref}}$, was measured by an ultrasonic anemometer permanently mounted in an upstream position, at $X = 5\,\hbox {m}$, $Y = 1\,\hbox {m}$, $z = 1\,\hbox {m}$, where *X* is the distance from the working-section inlet, *Y* is the lateral distance from the wind-tunnel centreline and *z* is the vertical distance from the wind-tunnel floor. There was negligible influence of the anemometer at the measurement stations. Control of the wind tunnel, of probe position, of the LDA and temperature-probe measurements and other data acquisition was provided by National Instruments LabView-based software.

## Results and Discussion

A list of all the cases is given in Table 1, where several quantities are defined below. While most measurements, the main sets, were made with the spires present, some were made in their absence—cases 1–6. Of these six, two were made with a smooth surface. Cases 7–21 correspond to a rough surface, and form three sets: uniform inlet temperature with full-floor cooling, variable $\Delta \Theta$; uniform inlet temperature with variation of the uncooled length of the floor, $X_{\mathrm{C}}$, constant $\Delta \Theta$; non-uniform inlet temperature profile and variable $X_{\mathrm{C}}$, constant $\Delta \Theta$. Note that $X_{\mathrm{C}}$ denotes the distance from the working-section inlet at which the surface cooling starts. (An uncooled length of floor was achieved by isolating the required number of panels from the chilled-water system.) The last seven cases, 16–22, were made at a reference velocity magnitude of $1.5\,\hbox {m\,s}^{-1}$, while the others were made at $1\,\hbox {m\,s}^{-1}$. This lower value was used in order to avoid larger temperature differences, and to reduce power requirements for the earlier runs. The Reynolds number ${ Re}_{h}$, defined as $U_h h/\nu$, where the kinematic viscosity, $\nu$, is that at the surface, is given in Table 1 as the range between the first and last measurement stations.

**Table 1:** Cases investigated and salient parameters at the *X*-stations given in figure legends

Case	Rough/smooth	Spires	$$\Delta \Theta $$ (K)	$$X_{\mathrm{C}}$$ (m)	*f*	$$L_0 $$ (m)	*h* (m)	$$Ri_h $$	$$Re_h \times 10^{-3}$$
1	S	N	0	–	–	–	0.16, 0.22, 0.30	–	22–21
2	R	N	0	–	–	–	0.20, 0.28, 0.38	–	13–26
3	S	N	10	1	Unif	0.61, 0.55, 0.44	0.15, 0.21, 0.27	0.046, 0.062, 0.077	11–20
4	R	N	5	1	Unif	1.26, 1.15, 1.14	0.17, 0.27, 0.30	0.029, 0.044, 0.046	12–21
5	R	N	10	1	Unif	0.72, 0.58, 0.48	0.17, 0.27, 0.34	0.052, 0.080, 0.095	12–25
6	R	N	19	1	Unif	0.31, 0.20, 0.16	0.18, 0.32, 0.47	0.098, 0.170, 0.243	13–35
7	R	Y	0	–	–	–	0.55, 0.55, 0.55	–	37–38
8	R	Y	5	1	Unif	0.97, 0.99, 0.99	0.55, 0.55, 0.60	0.090, 0.088, 0.092	38–43
9	R	Y	7.5	1	Unif	0.61, 0.64, 0.54	0.55, 0.60, 0.70	0.131, 0.141, 0.158	38–50
10	R	Y	10	1	Unif	0.43, 0.44, 0.31	0.52, 0.66, 0.84	0.161, 0.197, 0.243	36–60
11	R	Y	5	1	Unif	0.79, 0.89, 0.90	0.55, 0.55, 0.55	0.091, 0.085, 0.086	37–39
12	R	Y	5	3	Unif	0.86, 0.99, 1.00	0.55, 0.55, 0.55	0.087, 0.085, 0.081	37–39
13	R	Y	5	5	Unif	1.11, 0.94,0.81	0.55, 0.55, 0.55	0.085, 0.088, 0.083	37–39
14	R	Y	5	7	Unif	6.34, 0.97, 0.95	0.55, 0.55, 0.55	0.088, 0.086, 0.082	37–39
15	R	Y	0	–	–	–	0.55, 0.55, 0.55	–	37–39
16	R	Y	16	5	0 (Unif)	0.84, 0.62, 0.57	0.55, 0.55, 0.55	0.128, 0.126, 0.122	54–56
17	R	Y	16	5	1	1.73, 0.87, 0.81	0.55, 0.55, 0.55	0.128, 0.124, 0.120	55–57
18	R	Y	16	5	0.2	0.62	0.55	0.127	55
19	R	Y	16	5	0.4	1.26, 0.75, 0.65	0.55, 0.55, 0.55	0.128, 0.125, 0.121	55–56
20	R	Y	16	1	0.4	0.68, 0.64, 0.68	0.55, 0.55, 0.55	0.128, 0.125, 0.120	54–56
21	R	Y	16	3	0.4	0.68, 0.72, 0.68	0.55, 0.55, 0.55	0.128, 0.125, 0.121	55–55
22	R	Y	16	5	0.4	0.75, 0.65, 0.68, 0.68	0.55, 0.55, 0.55, 0.55	0.144, 0.142, 0.140, 0.138	55–56

The data in the columns for $$L_0 $$, *h* and $$Ri_h $$ correspond to the three measurement stations in *X* or, for case 22, the four stations. The right-hand-most column gives the range of $${ Re}_{h}$$ between the first and last measurement stations. ‘Unif’ in the column for *f* denotes a uniform inlet temperature profile

For the most part, measurements were made at three stations: $X = 6.4$, 10.7 and $15.0\,\hbox {m}$. The first station is where the flow is normally still developing downstream of the spires, and between this and the last station the freestream velocity increased by $3.0 \pm 0.6\%$. The last case, case 22, is based on a culmination of what was learnt from the preceding cases, and is presented in Sects. 4.4 and 4.5. For this case the measurements were made at $X = 9.2$, 10, 12.1 and 14.2 m, over which the freestream velocity increased by 1.8%; any effects of pressure gradient on turbulence structure are assumed to be negligible. ${ Re}_{h}$ for this case is about four times or more larger than that covered by Williams et al. (2017).

So as to be economical in the number of point measurements made, the vertical positions were spaced logarithmically, and as a result, the points near the upper edge of the boundary layer are relatively widely spaced. Rather than fitting curves to the profiles, the height, *h*, has been determined by eye as the point at which *U* has reached the freestream value, and suffices for the present purposes.

### No Spires

The profiles presented in this section are given in terms of *z* / *h* while for the later cases they are given in terms of *z*, and *U* is normalized by $U_{{h}}$, rather than $U_{{Ref}}$. The growth of the boundary-layer height can be seen from Table 1, and $U_{{h}}/U_{{Ref}}$ is given in Table 2.

**Table 2:** $U_{{h}}/U_{{Ref}}$ for cases 1–6, at the stations in *X*

Case	$X = 6.4$, 10.7, 15.0 (m)	Case	$X = 6.4$, 10.7, 15.0 (m)
1	1.011, 1.030, 1.051	4	1.018, 1.036, 1.061
2	1.014, 1.034, 1.053	5	1.044, 1.063, 1.083
3	1.045, 1.064, 1.083	6	1.092, 1.107, 1.121

**Fig. 2:**
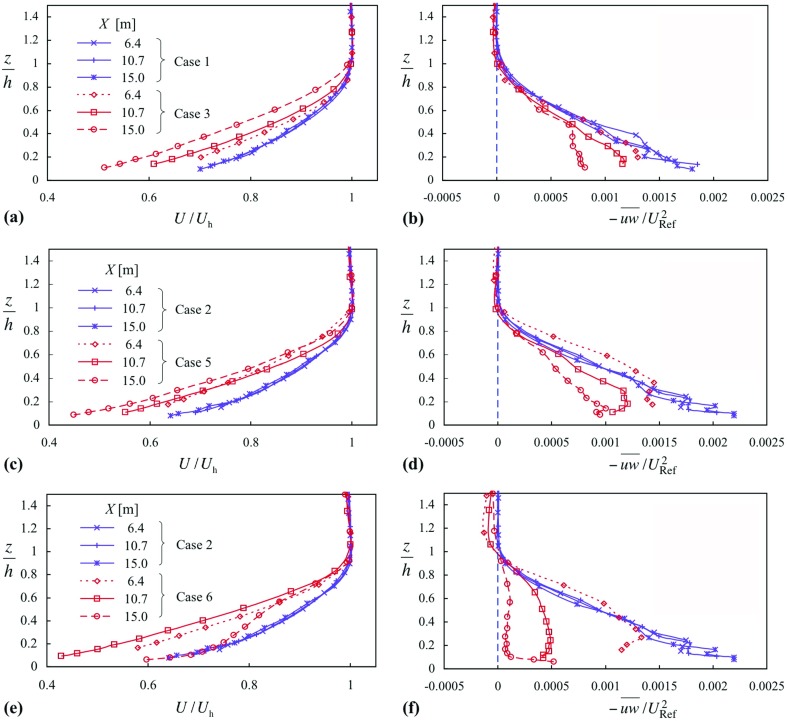
Profiles of mean velocity and Reynolds shear stress for three comparisons: **a**, **b** case 3 with respect to case 1; **c**, **d** case 5 with respect to case 2; **e**, **f** case 6 with respect to case 2. Symbols as in (**a**), (**c**) and (**e**). Cases in (**a**) and (**b**), smooth surface; others, rough

**Fig. 3:**
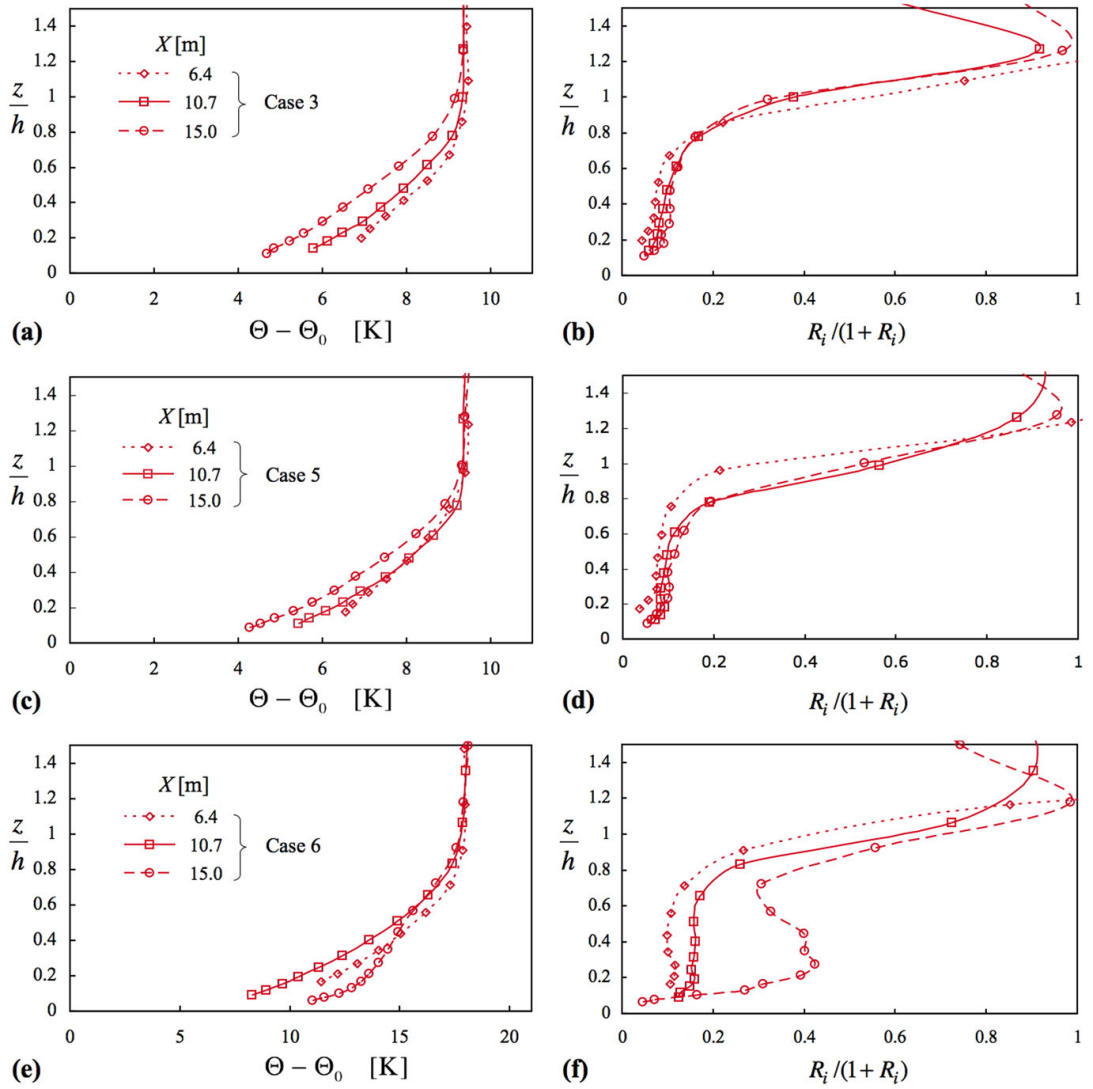
Profiles of mean temperature and Richardson number corresponding to Fig. 2: **a**, **b** case 3; **c**, **d** case 5; **e**, **f** case 6. Symbols as in (**a**), (**c**) and (**e**). Case in (**a**) and (**b**), smooth surface; in others, rough

Figures 2 and 3 give profiles of mean velocity, Reynolds shear stress, mean temperature and gradient Richardson number, though presented as $Ri/(1+Ri)$, for cases 3, 5 and 6; Fig. 2 also includes the respective neutral cases (1 and 2). A distinctive feature of the smooth-surface flow, case 3 (in Figs. 2a, b, 3a, b), is the change in the shape of the mean velocity profiles with increasing *X* (where ${ Ri}_{{h}}$ also increases), the ‘knee’ of the profile becoming progressively sharper (Fig. 2a). In addition, $-\,\overline{uw}$ (Fig. 2b) becomes substantially reduced in the lower half of the layer, while *Ri* (Fig. 3b) exhibits a plateau region in the centre of the layer, with a level that increases with *X*, before rising rapidly near the layer top. When the surface is rough, case 5 (Figs. 2c, d, 3c, d), the behaviour in the profiles of *U*(*z*) is different, where there is much less variation across the three stations for a slightly larger variation in ${ Ri}_{h}$ (Table 1). Again, $-\,\overline{uw}$ decreases and *Ri* increases with increasing *X*.

When ${ Ri}_{{h}}$ is increased further, as in case 6 (Figs. 2e, f, 3e, f), there is a dramatic change in the profile of *U*(*z*) between the second and third stations, a collapse in shear stress to almost zero over most of the layer, and a large increase in *Ri*. Taking note of the theoretical limit of ${ Ri }= 0.25$ (i.e. $Ri/(1+Ri) = 0.2$), the *Ri* values at the third-station profile are well above this limit. In the lower part of the boundary layer the profile of *U*(*z*) at the last station is coincident with that of the neutral layer (though this is very likely fortuitous), and the shear stress shows a sharp rise near the surface, consistent with large ${\partial {U}}/{\partial {z}}$ near the surface. The profile of mean temperature also undergoes a change rather like that seen in the mean velocity profile. The changes taking place between these two stations occur over a distance of about 10 h, and are worthy of further investigation themselves.

### With Spires, and Uniform Inlet Temperature Profile

#### $X_{\mathrm{C}}=1\,\hbox {m}$

**Fig. 4:**
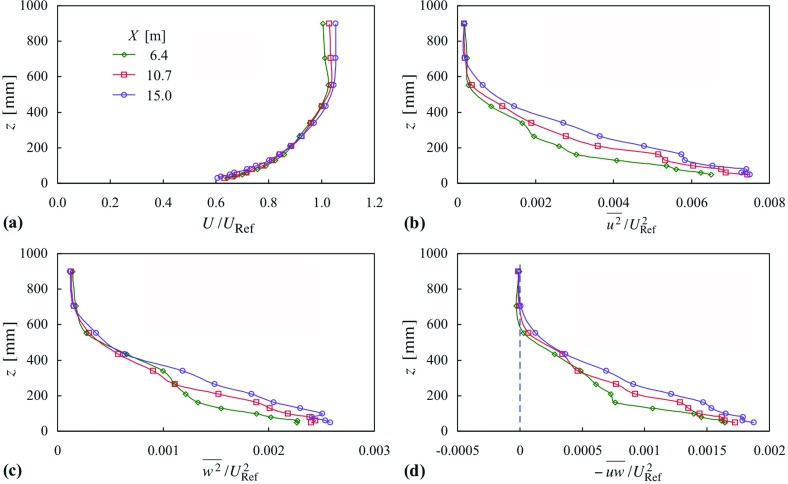
Profiles of mean velocity and Reynolds stress, at three stations downstream of the spires, in neutral flow, case 7. Symbols as in (**a**)

Figure 4 shows profiles of mean velocity; and Reynolds stresses for neutral flow, for the same three streamwise stations as in Fig. 2. In contrast, the mean velocity profiles in Fig. 4 differ from each other only slightly (and do so most noticeably in the freestream as a result of a small negative pressure gradient), where the boundary-layer height has been taken as the same in each case (as given in Table 1). The shape of the mean velocity profiles, taken together, is clearly different from those in Fig. 2. The profiles of each respective Reynolds stress in Fig. 4 are comparable, though there is clearly development in each. (The differences between the stations would be less if *z* where normalized on a height based on the shear stress and the local freestream velocity.) As noted earlier, profiles at the first station are in the region where the flow is still undergoing development downstream of the spires. The second and third profiles are more closely comparable in each instance.

**Fig. 5:**
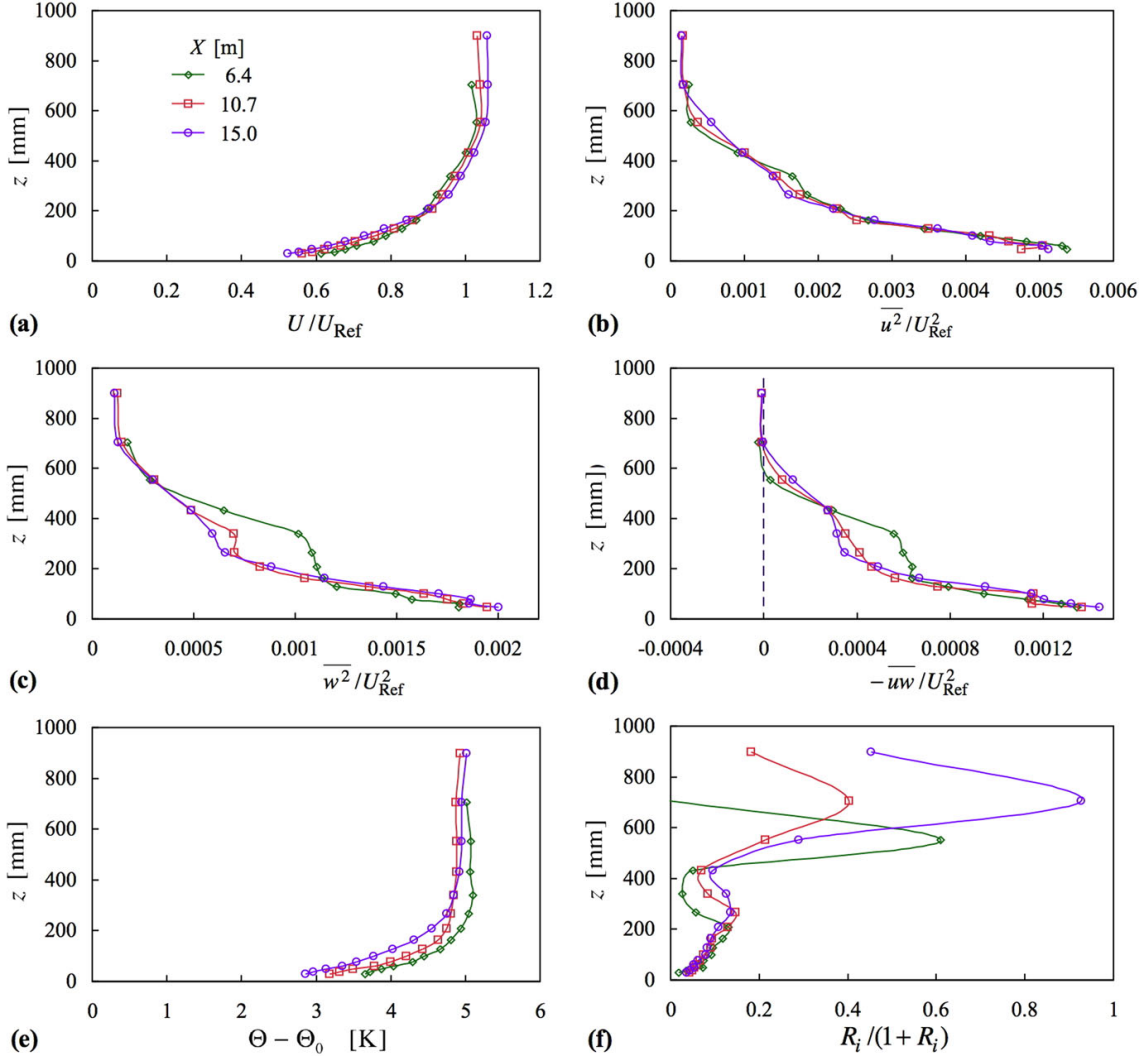
Profiles of mean velocity, Reynolds stress, mean temperature and Richardson number, at three stations in stable flow, case 8 (uniform inlet temperature profile). Symbols as in (**a**)

**Fig. 6:**
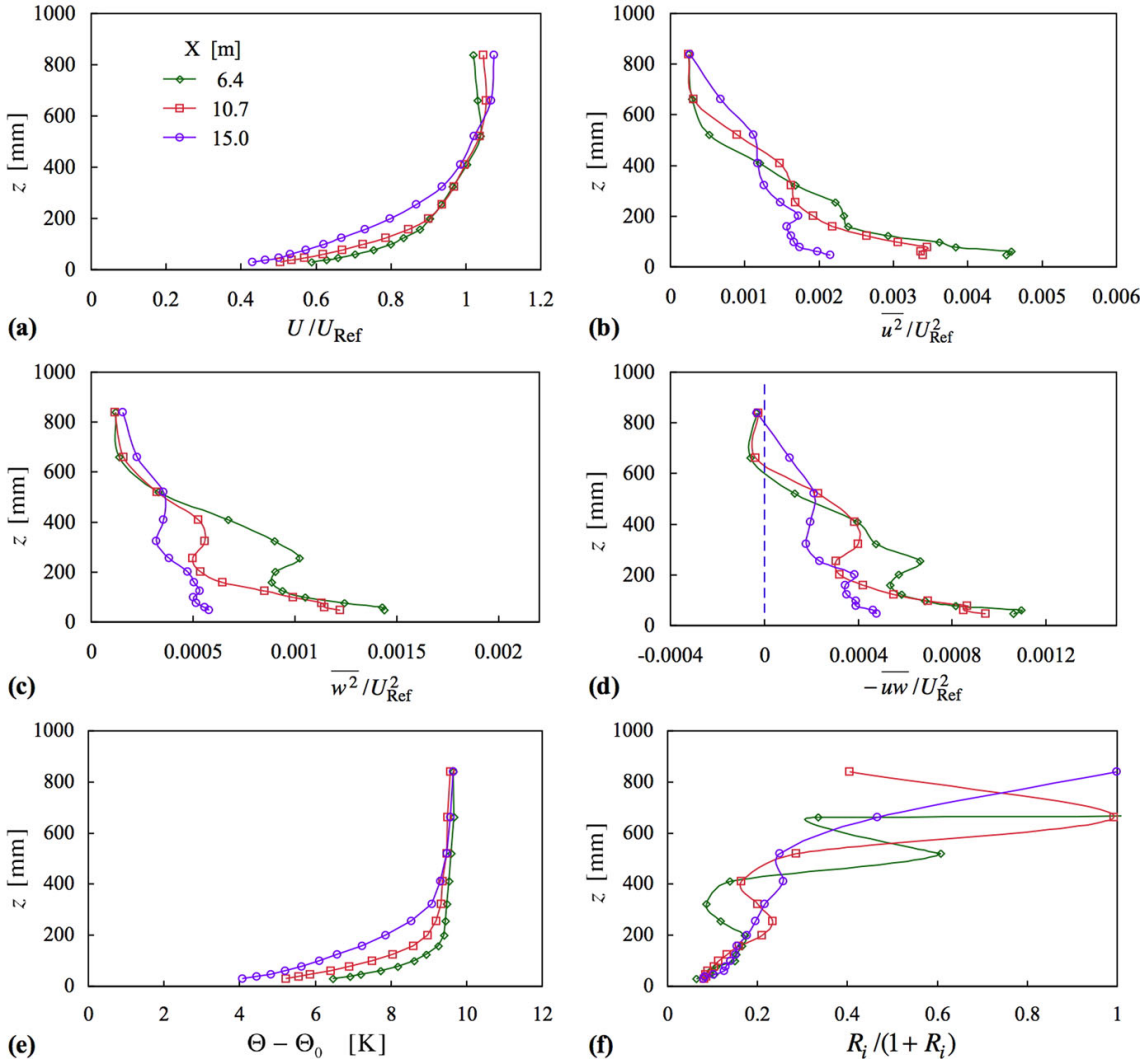
Profiles of mean velocity, Reynolds stress, mean temperature and Richardson number, at three stations in stable flow, case 10 (Uniform inlet temperature profile). Symbols as in (**a**)

Figures 5 and 6 give profiles of momentum and thermal quantities for two cases, 8 and 10, for two temperature differences, $\Delta \Theta = 5$ and 10 K, respectively. Taking case 8 first, where ${ Ri}_{h}$ is about 0.09, the mean velocity profiles are close to each other, and close in shape to those of case 7 (Fig. 4). Interestingly, the profiles of $\overline{u^{2}}$ fall close to each other, much more so than in the isothermal case. The same is not true, though, for either $\overline{w^{2}}$ or $-\overline{uw}$, where there is clear development taking place in the middle of the layer between the first and second stations (and perhaps, in fact, a residual amount for $\overline{u^{2}} )$, and a residual amount between the second and third. Above and below this ‘middle zone’ the profiles compare more closely. Another point to note from Fig. 5b is that the freestream turbulence intensity, $\overline{u^{2}}^{1/2}/U$, is 1.2% compared with about 4% in Hancock and Pascheke (2014).

Comparison of the mean temperature and Richardson number profiles with those of Fig. 3 shows clear differences. In Fig. 3 it is unmistakable that the mean temperature varies across the whole height of the layer, whereas in Fig. 5 significant variation exists only over the lower half of the layer, and the upper half is at very nearly constant temperature, implying near-neutral flow. It appears the layer can be categorized as having an upper part—about the upper 1/3—as a near-neutral flow; a lower part—about the lower 1/3—as a stable flow, where *Ri* and other quantities suggest surface-layer-like behaviour; and an ‘adjustment region’ in between.

In case 10 (Fig. 6) the temperature difference is twice that of case 8. (Case 9 of intermediate difference is not shown. The profiles are more like those of case 8 than they are of case 10.) Here, in Fig. 6, there is much less comparability between the profiles of each quantity. The mean velocity profile at the downstream station is clearly different from those further upstream, though not in the way seen for case 6 in Fig. 2e. The height (*h*) has increased, as also seen from the Reynolds stress profiles. There is a marked reduction in $-\,\overline{uw}$, but no dramatic reduction as there was in case 6. The development in *U*(*z*) that takes place between the first and second stations of case 8 can also be seen here, but the strength of the stability is such that the near invariance seen between the second and third stations (of case 8) is not repeated. Again, from the mean temperature profiles, the upper 1/3 is a neutral flow, or nearly so.

#### $\Delta \Theta = 5\,\mathrm{K}$, *Effect of*$X_{\mathrm{C}}$

**Fig. 7:**
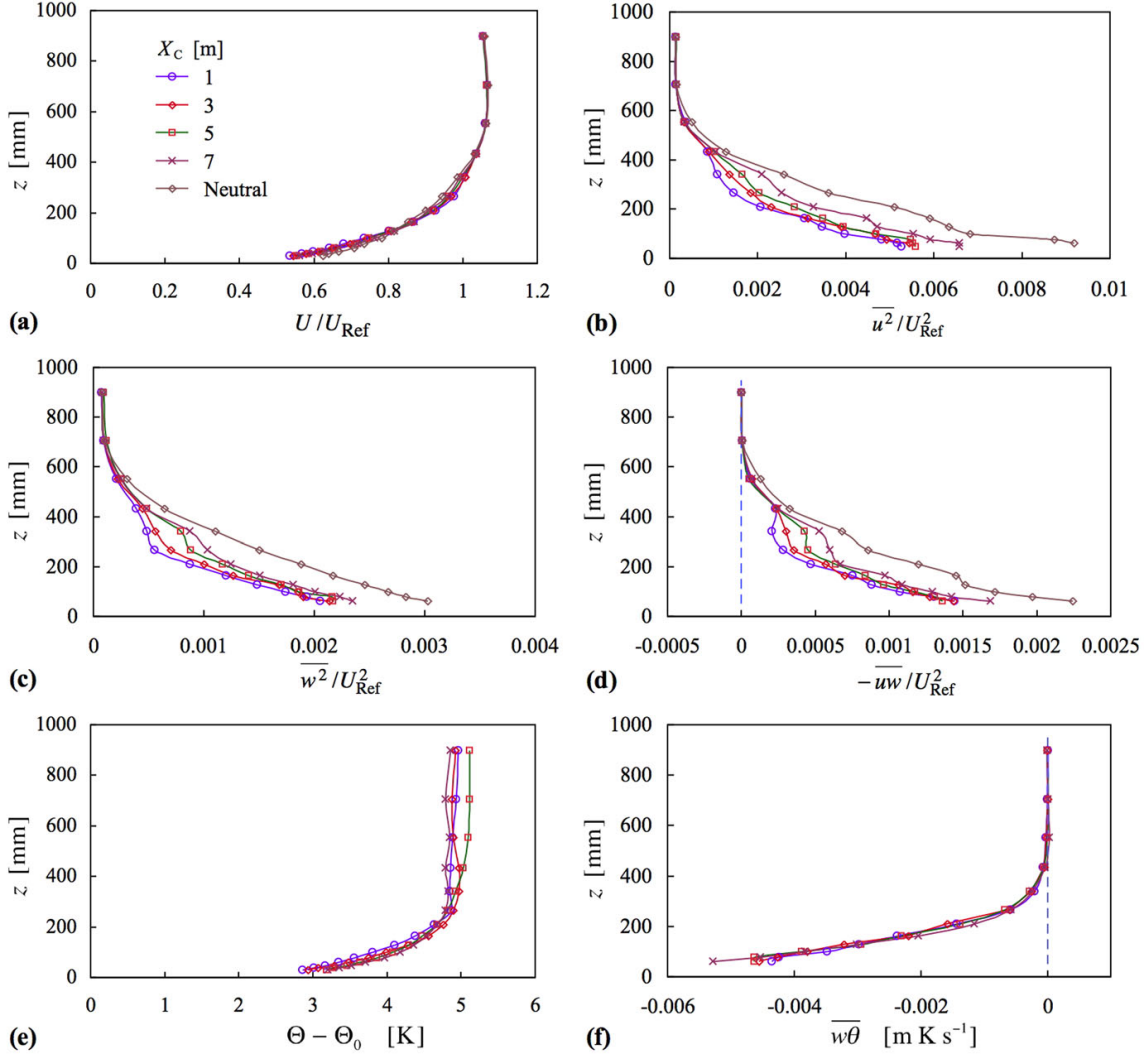
Profiles of mean velocity, Reynolds stress, mean temperature and heat flux, at $X = 15\,\hbox {m}$ as a function of uncooled floor length, cases 11–14, and neutral flow (case 15) (uniform inlet temperature profile). Symbols as in (**a**)

In the foregoing the length of the cooled floor was constant, and the temperature difference, $\Delta \Theta$, was varied; now, $\Delta \Theta$ is constant, and the length of uncooled floor, $X_{\mathrm{C}}$, is varied. Figure 7 shows momentum and thermal quantities for $X_{\mathrm{C}} = 1$, 3, 5 and 7 m, cases 11–14, where the (kinematic) vertical heat flux $\overline{w\theta }$ is shown rather than *Ri*. (Profiles of *Ri* are very much like those in Fig. 5.) The first (case 11) is nominally a repeat of case 8, but the conditions were slightly different, and so is retained here.^2^

Figure 7 shows how varying $X_{\mathrm{C}}$ affects the profiles at a single station ($X = 15\,\hbox {m}$), for all four values of $X_{\mathrm{C}}$; this figure also includes case 15, for isothermal flow. The effect on the mean velocity is slight, but the influence on the Reynolds stresses is very clear, in all three stresses. As the uncooled length is increased the reduction in the stresses is lessened, and each of the profile sets shows that the upper 1/3 is little or not affected but, curiously, at the same time retaining a distinct difference from the profiles of the neutral flow. It is supposed a greater uncooled length results in a still smaller reduction, and that the profiles fall closer to those of the neutral set. Again, it appears that the layer may be thought of as having three parts, with the lower 1/3 being controlled by the length of uncooled floor, and the middle 1/3 being an adjustment region. The trend in the upper 1/3, that each of the Reynolds stress profiles falls below that of the respective neutral profile, is seen particularly clearly in $-\,\overline{uw}$. It does seem odd, however, that the structure of the boundary layer changes below this upper 1/3 (as indicated by the reduction in the Reynolds stresses) without there being any effect above, because, at least for neutral flow, the eddy sizes are of the order of the height of the layer. It is certainly a matter for further investigation. Finally, although there is significant variation in Reynolds stresses, there is little variation in the profiles of mean temperature and virtually none in those of heat flux ($\overline{w\theta }$). Given the reductions in $\overline{w^{2}}$ and $-\,\overline{uw}$ this lack of variation is also rather curious.

**Fig. 8:**
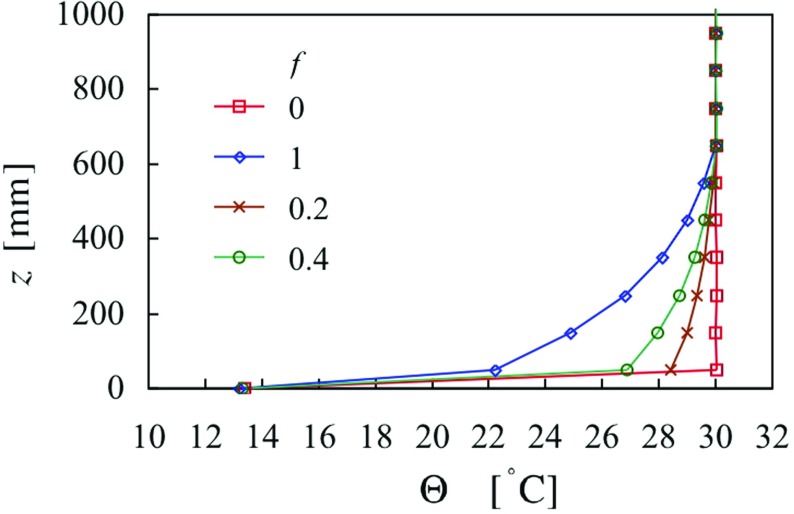
Inlet temperature profiles as a function of the factor *f*. The surface temperature (at $z = 0\,\hbox {mm}$) is also shown for each case, but is independent of *f*

### With Spires, and Non-uniform Inlet Temperature Profile

In the early stages a number of trials was undertaken in which a non-uniform profile with an overlying inversion was tried, but it was difficult to understand the results. Not imposing an inversion simplified matters in one regard. However, given the foregoing results, it is clear that the inlet profile must be non-uniform if the whole depth of the layer is to be stable. Employing the iterative strategy that had worked for a convective layer (Hancock et al. 2013) was also unsatisfactory, not that surprisingly, since the temperature profile did not extend the full height in the first place (with a uniform inlet profile). Therefore, it was decided to use a profile from case 4 where, in the absence of the spires, the mean temperature profile extends the full depth of the layer. The profile, scaled to the height of the ‘with spires’ layer and to the chosen temperature difference, is shown in Fig. 8, along with three other cases. One of these three is just the uniform inlet profile, and all are given by

7 $$\begin{aligned} \Theta (z)=\Theta _h -f\left[ {\Theta _h -\Theta (z)} \right] , \end{aligned}$$

where the square brackets denote the original scaled case. $\Delta \Theta =\Theta _h -\Theta _0$ is fixed (at the chosen difference), and *f* is a variable between $f = 0$ and 1, where $f = 0$ gives the uniform profile, and $f = 1$ gives the (scaled) profile from case 4. Figure 8 includes profiles for $f = 0.2$ and 0.4. There is still a large jump between the temperature specified for the lowest inlet heater and that specified for the surface, but this was deemed acceptable because the gradient in temperature is in any case very large near the surface.

**Fig. 9:**
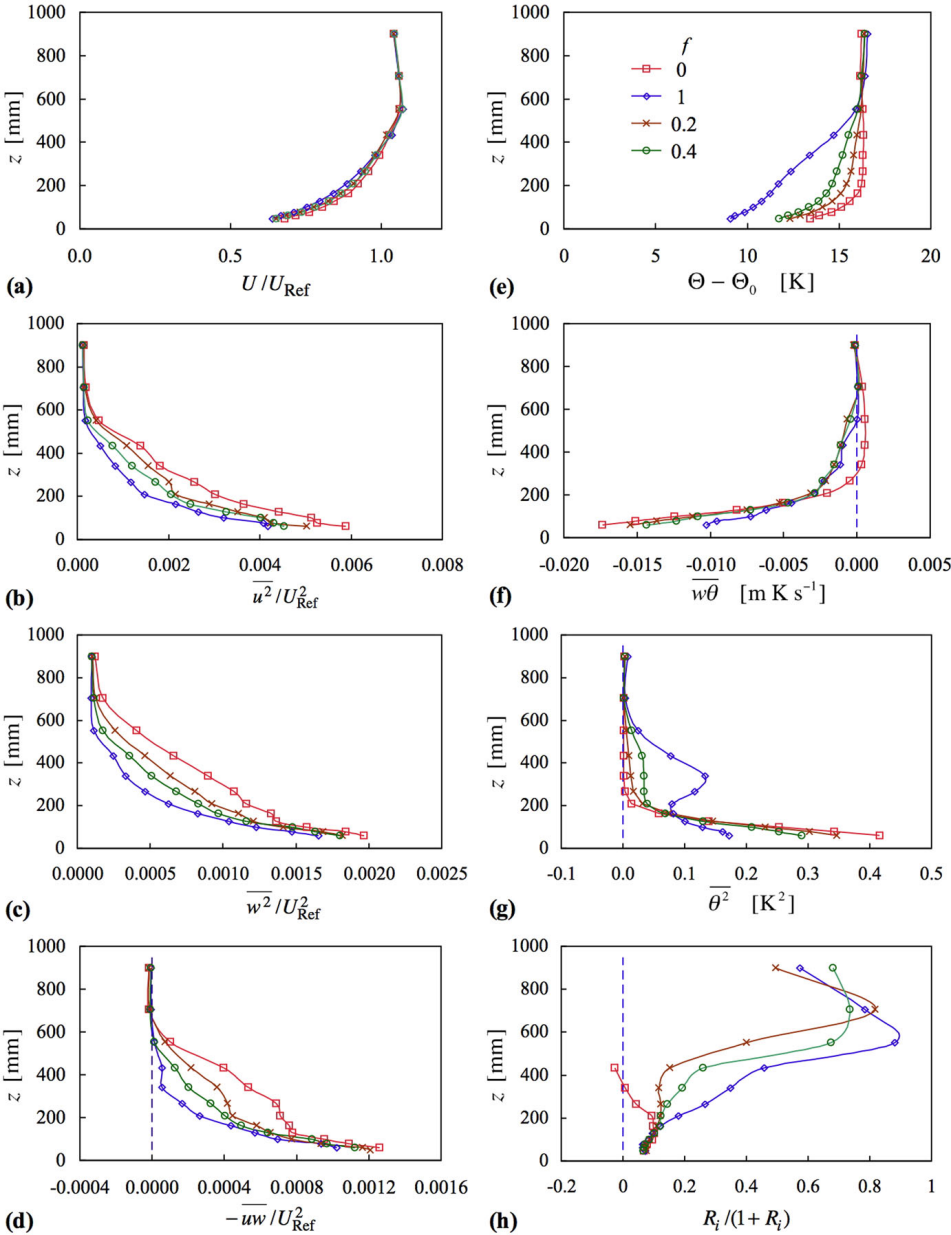
Profiles of mean velocity, Reynolds stress, mean temperature, heat flux, mean-square temperature fluctuation and Richardson number, at $X = 10.4\,\hbox {m}$, as a function of inlet temperature profile parameter, *f*. Cases 16–19. Symbols as in (**e**)

Figure 9 shows momentum and thermal quantities at $X = 10.4\,\hbox {m}$, for the four cases of Fig. 8 (cases 16–19), for which $X_{\mathrm{C}}$ was fixed at 5 m for each. Again, the mean velocity profiles show only a small variation. The Reynolds stresses show a systematic development to progressively more concave shapes as *f* increases from 0 to 1. They are lowest in magnitude in the centre of the layer, at, say, $z = 300\,\hbox {mm}$, when $f = 1$. Profiles of mean temperature show steady increases up to the edge of the layer, except for the case $f = 0$, the uniform inlet case. Thus this strategy achieved at least one main task, that the temperature should rise throughout the depth of the layer.

The value of *f* clearly has a marked effect on the mean square of the temperature fluctuation, $\overline{\theta ^{2}}$, the case $f = 1$ leading to $\overline{\theta ^{2}}$ falling and then rising to a peak in about the centre of the layer, before falling to zero at the top edge. The peak was larger at the upstream station and smaller at the downstream station (neither shown). A high level in the centre of the layer is not so surprising, given the gradient in mean temperature in the presence of vertical velocity fluctuations, which decrease to a low magnitude at the top of the layer. More surprising is the minimum beneath the peak. The value of *f* = 0.4 leads to a plateau rather than a peak.

The effect on the vertical heat flux, $\overline{w\theta }$, is mixed; below about $z = 200\,\hbox {mm}$, the profiles for $f = 0$, 0.2 and 0.4 coincide closely, while above this height, those for $f = 0.2$, 0.4 and 1 coincide closely. The reduced flux near the surface for $f = 1$ is perhaps to be expected because the temperature difference imposed between the lowest inlet heater and the surface is the lowest of all (see Fig. 8), and is more marked in this instance. Overall, though, the trend in heat flux near the surface follows the trend in the near-surface temperature.

It is not expected that the profiles of the various quantities presented in this section should match those of profiles obtained when no spires were present, even though a temperature profile of the latter has been ‘borrowed’. For one thing, the mean velocity profiles are different and remained mostly unaffected across the various cases. However, it is interesting to note that the profile for *Ri*, for the case $f = 0.2$ is closest to the profiles given in Fig. 3d. It is envisaged that in due course a suitable parametrization for the inlet profile, beyond that employed here, could be used to ‘fine tune’ the profiles of *Ri* and other quantities, for a given set of generators. Different generators would give different profiles of mean velocity and other quantities.

Though not shown here, varying $X_{\mathrm{C}}$ had a similar effect to that seen in Fig. 7, except that there was also an effect seen in the vertical heat flux in the middle 1/3 of the layer.

### Example ‘Final Case’

**Fig. 10:**
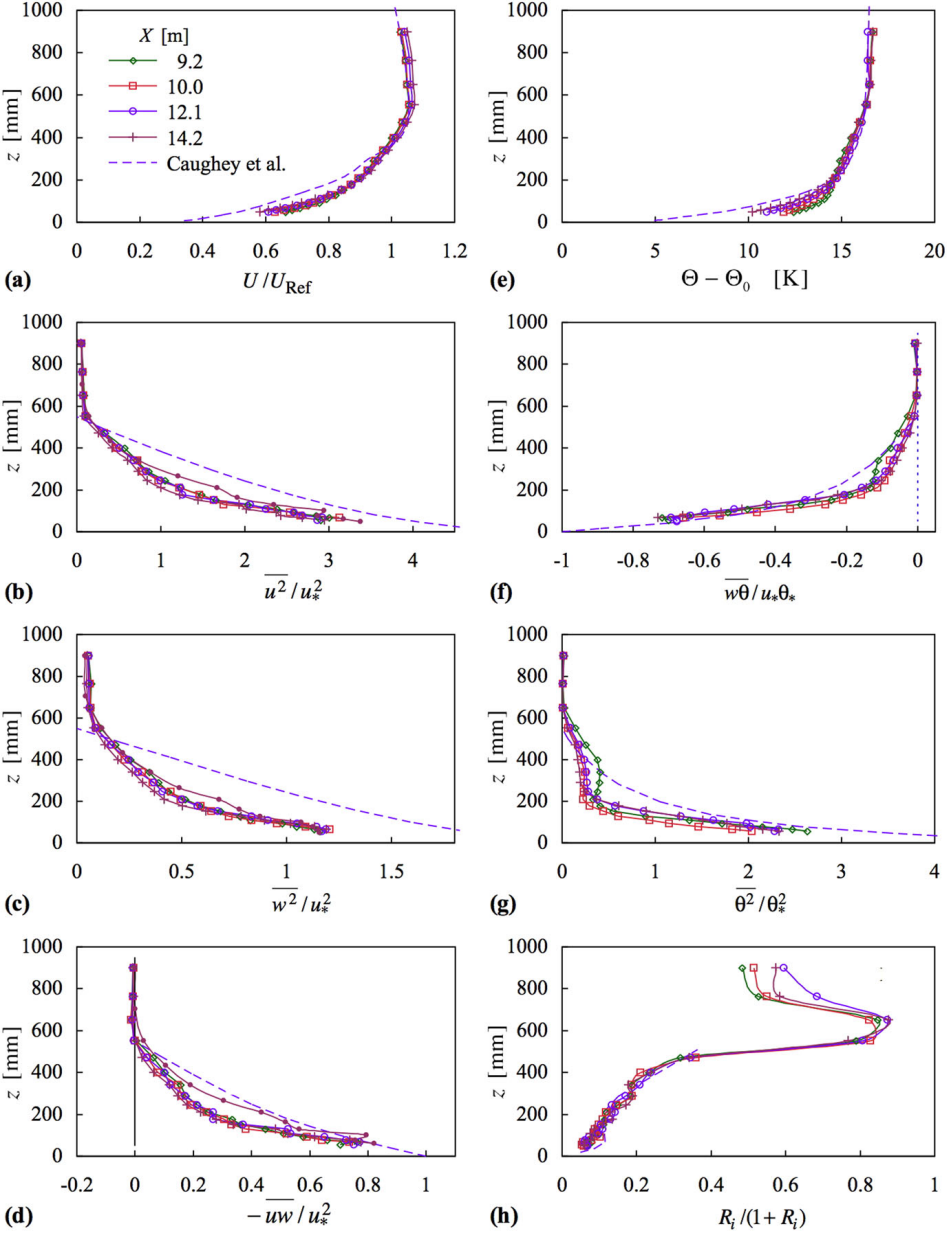
Profiles of mean velocity, Reynolds stresses, mean temperature, heat flux, mean-square temperature fluctuation and Richardson number, at four stations in *X*. Case 22. Symbols as in (**a**). The full lines with dots in the Reynolds stress profiles are for neutral flow at $X = 10.7\,\hbox {m}$ (for which $u_*/U_h = 0.046$; see Table 3 for stable profiles). The broken line in each is based on Caughey et al. (1979); see text

As stated earlier, the objective in using tall flow generators is to create a deep boundary layer (at about the height of the generators) that does not vary greatly in the streamwise direction over a distance of interest in question, and that has selected characteristics of the ABL. In the foregoing, the measurements at the first station ($X = 6.4\,\hbox {m}$) are still in the development region. A further set of measurements was made at four stations between $X = 9.2$ and 14.2 m—i.e. over a distance of about 9 h. These are shown in Fig. 10, and as can be seen each of the various quantities does not vary much with streamwise position, implying approximately horizontally homogeneous flow over this fetch. Note, here, the second-order moments are normalized with respect to surface layer quantities, where $\theta _{*} =-(\overline{w\theta } )_0 /u_*$. Normalized instead by the reference velocity, the Reynolds stress profiles would show only a very slightly larger spread. Such normalization is necessary for assessing the degree to which horizontal homogeneity is or is not achieved.

On the other hand, normalization by the surface shear stress allows, for example, comparison with the Reynolds stress profiles of the neutral flow. For very weak stability it is to be anticipated that the Reynolds stress profiles over the full height of the layer would still scale on $$u_*^2 $$, but not as the stability increases, except in the surface layer, and not even then for very stable conditions (e.g. Sorbjan 2010). Figure 10b–d include profiles for neutral flow. They indicate that the turbulence over most of the layer is relatively less strong in the stable case, though evidently not by a large amount at this level of stability. The most marked reduction is in the shear stress. Near the surface, however, there is little to distinguish the stable and neutral profiles, consistent with surface-layer scaling applying. The Reynolds stresses of the stable flow are, of course, lower than in the neutral case, in terms of $$U_{h}$$, say. The friction velocity was about 18% lower in this example.

Figure 10 also shows curves taken from Caughey et al. (1979). Those for the second moments have been taken from the curves given in their Fig. 5, while those for mean velocity and mean temperature have been taken from their Fig. 2, which they describe as typical. The curve given in Fig. 10a follows straightforwardly from similarity, adjusted to match the maximum in $U/U_{{Ref}}$. That for the mean temperature (Fig. 10e) has been obtained from similarity of gradient Richardson number, *Ri*. Integration of $\partial \Theta /\partial z$ leads to

8 $$\begin{aligned} \Theta _h (z)-\Theta _h (h)=-\frac{\Theta _h }{\Theta _H }\frac{H}{h}\left( {\frac{U_{{Ref\,h}} }{U_{{Ref\,H}} }} \right) ^{2}\left( {\Theta _H (H)-\Theta _H (z)} \right) , \end{aligned}$$

where *H* is the boundary-layer height at full scale, and also denotes full scale, while *h* denotes laboratory scale. The integration is downwards, from the freestream, and the curve in Fig. 10e has been set to meet the freestream value.

The mean velocity profiles from the simulation, Fig. 10a, are in fact quite close to the profile given by Caughey et al. (1979), though with the simulated profiles being fuller in the lower half of the layer. (The near concurrence above the top of the layer with the small negative slope is fortuitous, arising from a slight non-uniformity in the wind-tunnel freestream flow.) Given the sensitivity of the boundary layer to the shape of the inlet temperature profile, the closeness of the (scaled) temperature profile to the profiles of the simulated layer is quite fascinating—and satisfying. The full-scale profile is slightly more rounded in the central part of the layer. The profiles at the four stations highlight the fact that there is still some development taking place near the surface but not further out.

The Reynolds stress profiles fall below those given by Caughey et al. (1979), most notably for $\overline{w^{2}}$. However, measurements subsequent to those given here by Marucci et al. (2016) for a higher surface roughness, more typical of rural terrain, show closer agreement for $\overline{u^{2}}$ and $-\,\overline{uw}$ (though still with a large discrepancy for $\overline{w^{2}} )$. The quantities $\overline{w\theta }$ and $\overline{\theta ^{2}}$ at the four stations have profiles that are less smoothly varying with height than found by Caughey et al. (1979), and $\overline{\theta ^{2}}$ shows a streamwise variation in the lower part of the layer that is presumably linked to the streamwise variation seen in the mean temperature profiles. Even so, the behaviour near the surface is quite close to that given by Caughey et al. (1979). If $\overline{\theta ^{2}}$is not normalized by $\theta _*$ the profiles are much closer to each other above $z = 200\,\hbox {mm}$, but a little more widely spread below.

Rather impressively, the gradient Richardson number implied by Fig. 2 of Caughey et al. (1979) falls on top of the closely coincident curves from the four stations, as can be seen in Fig. 10h. (Above $z = 500\,\hbox {mm}$, once scaled, there are too few field measurements, and $\partial U /\partial z$ passes through zero. Note, the concurrence seen in Fig. 10h is not a necessary condition for Eq. 8, which is a similarity scaling for mean temperature from full to laboratory scale.) Very clearly, the profile shapes and the behaviour seen in Fig. 10 are quite different from that seen in Fig. 2.

The analysis of Nieuwstadt (1985) predicts a linear decrease of turbulent heat flux, and variations of shear stress and other quantities according to $\overline{w^{2}} =2.0u_{*}^{2}(1-z/h)^{3/2}$, $\overline{uw} =u_{*}^{2}(1-z/h)^{3/2}$ and $\overline{\theta ^{2}} =\theta _{*}^{2}(1-z/h)^{1/2}$. The measurements in Fig. 10 do not follow these forms at all closely, and the closure assumptions of constant gradient and flux Richardson numbers are not met.

**Fig. 11:**
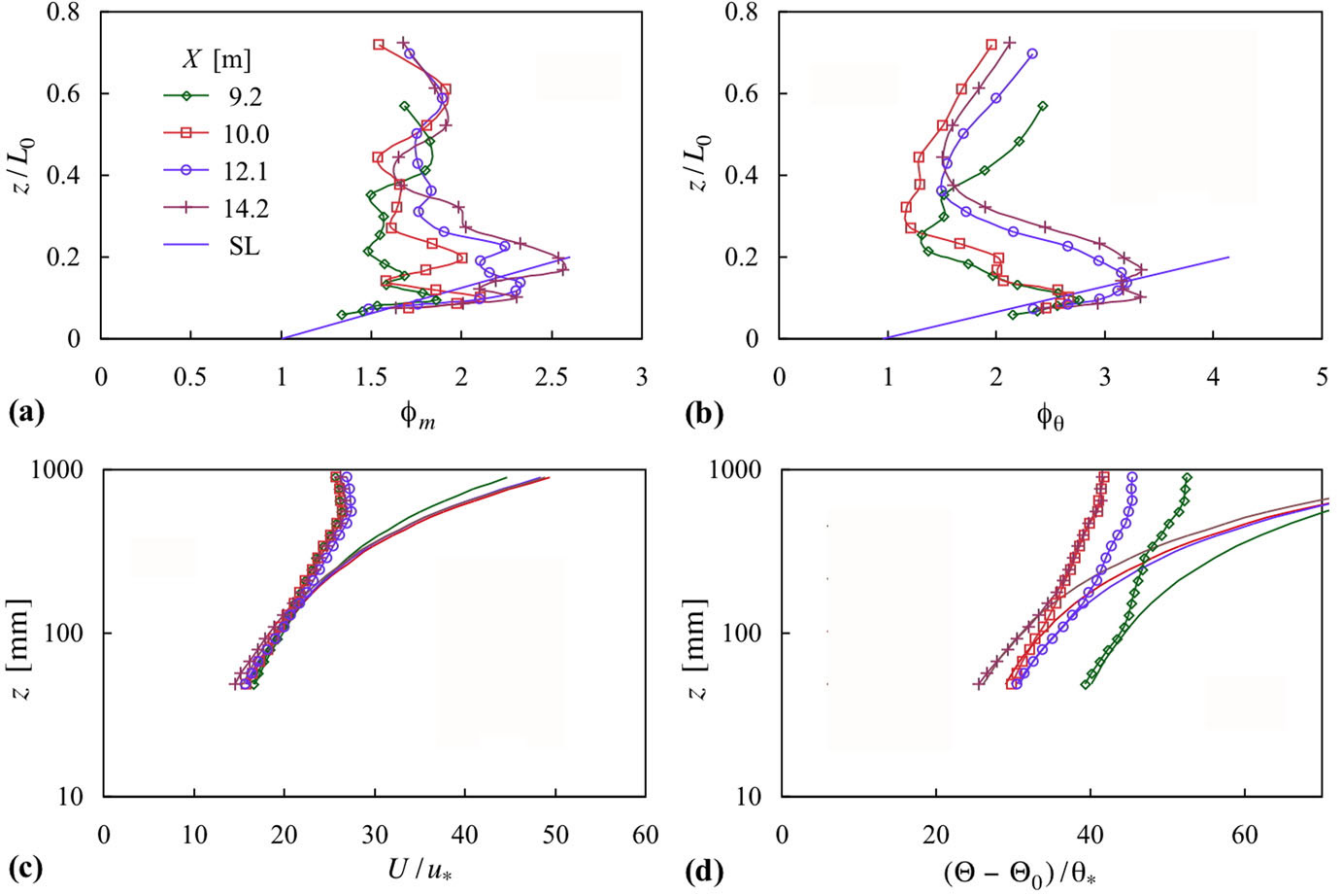
Surface-layer similarity functions, $\phi _m$ and $\phi _\theta$, mean velocity and mean temperature profiles for case 22. Symbols as in (**a**). SL denotes surface layer: lines in (**a**) and (**b**) are Eqs. 11 and 12, respectively. Lines in (**c**) and (**d**) are Eqs. 13 and 14

Figure 11 shows the surface-layer similarity functions for momentum and temperature, respectively, $\phi _m$ and $\phi _\theta$, where

9 $$\begin{aligned} \phi _m\equiv & {} \frac{\kappa z}{u_*}\frac{\partial U}{\partial z}, \end{aligned}$$

10 $$\begin{aligned} \phi _\theta\equiv & {} \frac{\kappa z}{\theta _*}\frac{\partial \Theta }{\partial z}, \end{aligned}$$

and where $\kappa$ is taken as 0.4. These figures also show the surface-layer forms according to

11 $$\begin{aligned} \phi _m= & {} 1+\beta _m \frac{z}{L_0 }, \end{aligned}$$

and

12 $$\begin{aligned} \phi _\theta= & {} 0.95+\beta _\theta \frac{z}{L_0 }, \end{aligned}$$

where Högström (1988) concludes $\beta _m = 5$ and $\beta _\theta = 8$ best represent quite scattered data. However, while the near-surface profiles of $\phi _m$ and $\phi _\theta$ converge to an approximately linear variation with *z*, rather larger constants are required in the above equations to match the measurements. Fits of the mean velocity and temperature measurements to, respectively,

13 $$\begin{aligned} U=\frac{u_*}{\kappa }\left[ {\ln \left( {\frac{z}{z_0 }} \right) +\beta _m \frac{z-z_0 }{L_0 }} \right] \end{aligned}$$

and

14 $$\begin{aligned} \Theta -\Theta _0 = \frac{\theta _*}{\kappa }\left[ {0.95 \ln \left( {\frac{z}{z_{0\theta } }} \right) +\beta _\theta \frac{z-z_{0\theta } }{L_0 }} \right] , \end{aligned}$$

are also shown in Fig. 11, and indicate that better fits are obtained with $\beta _m = 8$ and $\beta _\theta = 16$, corresponding to the upper end of the scatter given in Högström (1988). A value of $\beta _m = 8$ providing a better fit was noted by Hancock and Pascheke (2014), and a value of $\beta _\theta = 16$ would also match their measurements. Supposing the surface layer extends at most to a height of 0.15 *h*, the top of the surface layer will be at $z/L_0 = 0.12$ (i.e. $z = 83\,\hbox {mm}$) for these measurements. There are, therefore, only a small number of points in the surface layer, too few for a proper assessment. Shah and Bou-Zeid (2014) suggest from their *DNS* that $\beta _m$ increases at low Reynolds number, which may account for the high value. Their highest Reynolds number reached about 0.4 of that here: they obtained $\beta _m \approx 9$. Table 3 gives the values for the aerodynamic and thermal roughness lengths, and the surface-layer velocity and temperature scales employed in Fig. 11. Although, as regards the roughness elements, the set-up was very nearly^3^ the same as in Hancock and Pascheke (2014) and Hancock et al. 2013, the present values for these two quantities are more varied. It is not known why this is so, and a more detailed investigation is needed, but does not adversely affect the broader conclusions presented here, as the primary concern is the flow above the surface layer. The thermal roughness length is very much smaller than the aerodynamic roughness length, although a much smaller length has been observed repeatedly (see, for instance, Beljaars and Holtslag 1991; Duynkerke 1999), and was also observed by Hancock and Pascheke (2014).

**Table 3:** Surface-layer quantities, case 22

*X* (m)	$z_{0}$ (mm)	$z_{0\theta }$ (mm)	$u_*/U_h$	$\theta _*$ (K)
9.2	0.10	0.000006	0.038	0.32
10.0	0.15	0.0009	0.038	0.40
12.1	0.15	0.0004	0.036	0.36
14.2	0.19	0.0030	0.037	0.40

### Comparisons with the Local-Scaling Analyses of Nieuwstadt and Sorbjan

**Fig. 12:**
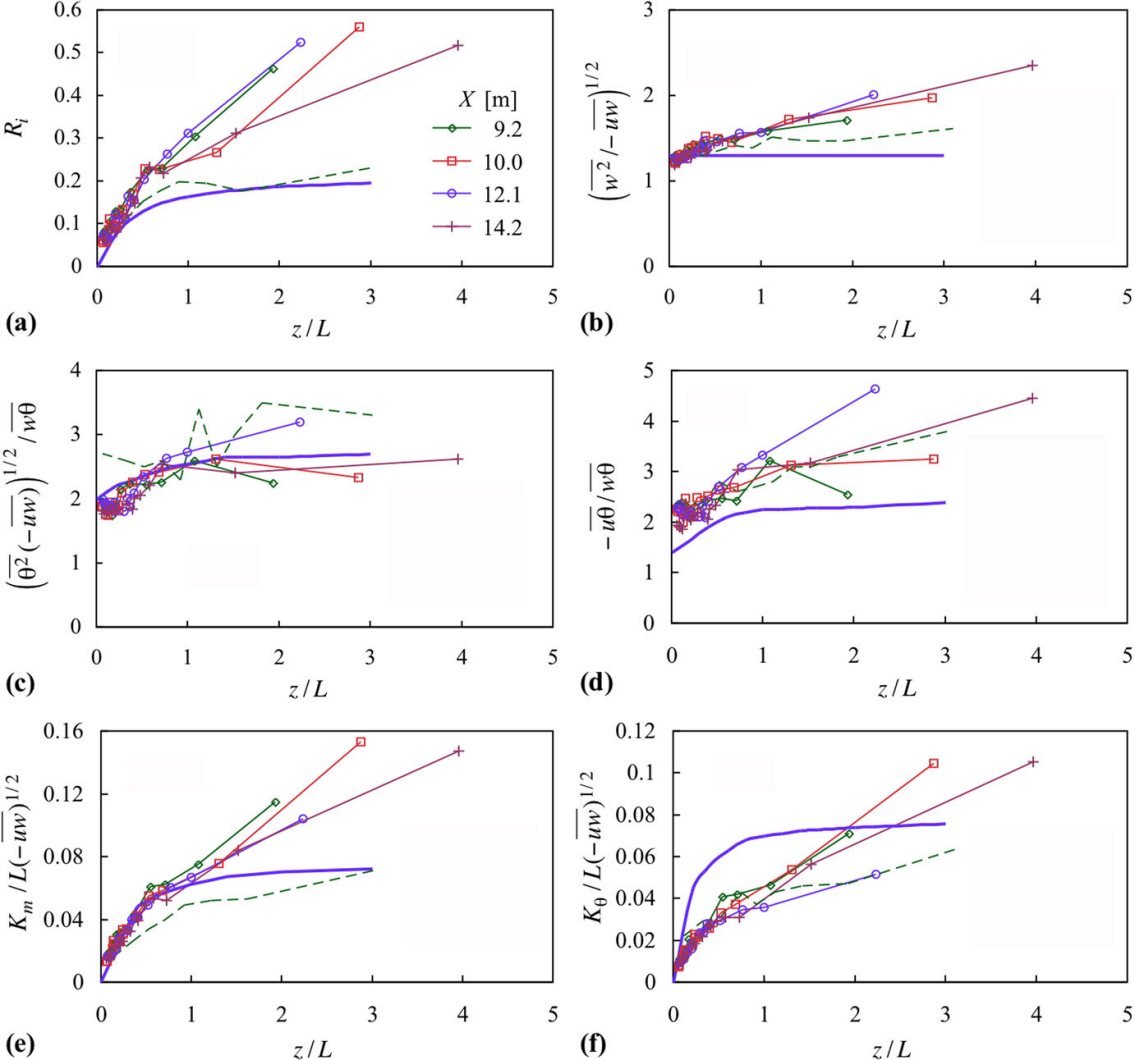
Parameters according to Nieuwstadt’s (1984) local scaling, for case 22. Symbols as in (**a**). Full lines show Nieuwstadt’s analytical results; broken lines, the field measurements

Case 22 is compared with the predicted behaviour according to the local scaling given by Nieuwstadt (1984) in Fig. 12, which shows the following quantities as functions of *z* / *L*

$$\begin{aligned} { Ri}, \frac{\overline{w^{2}}^{1/2}}{(-\overline{uw} )^{1/2}}, \frac{(\overline{\theta ^{2}} (- \overline{uw} ))^{1/2}}{\overline{w\theta } }, \frac{-\overline{u\theta } }{\overline{w\theta } }, \frac{K_m }{L (-\overline{uw} )^{1/2}}, \frac{K_\theta }{L (-\overline{uw})^{1/2}}, \end{aligned}$$

where

15 $$\begin{aligned} K_m\equiv & {} -\overline{uw} /\frac{\partial U}{\partial z}, \end{aligned}$$

16 $$\begin{aligned} K_\theta\equiv & {} -\overline{w\theta } /\frac{\partial \Theta }{\partial z} \end{aligned}$$

and

17 $$\begin{aligned} L \equiv -\frac{1}{\kappa }\frac{\Theta }{g}\frac{(-\overline{uw} )^{3/2}}{\overline{w\theta } }, \end{aligned}$$

the local Obukhov length. Local scaling implies that these quantities are each a function of *z* / *L* alone. In each set the four profiles fall close together, each indicating a plausible functional dependence on *z* / *L* alone, a point Nieuwstadt appealed to in his argument for local scaling and analysis of the Cabauw field data. *Ri* lies above the field data, which is fairly close to his theoretical curve. The ratio $(\overline{w^{2}} /(-\overline{uw} ))^{1/2}$ also lies above the theoretical curve, but is closer to the field data. The heat-flux ratio, $-\overline{u\theta } /\overline{w\theta }$, while above the theoretical curve, lies on the field data. The quantity $(\overline{\theta ^{2}} (- \overline{uw} ))^{1/2}/\overline{w\theta }$ falls close to the theoretical curve. Here, $K_{m}$ (non-dimensionalized as above) falls on the theoretical curve for a part of it, differing from the field measurements which fall below the curve. $K_\theta$ falls below the theoretical curve but concurs with the field measurements.

These quantities for the other cases generally show a larger variation from one profile to another than in Fig. 12 (after discounting measurements at the first station, $X = 6.4\,\hbox {m}$), and do not indicate dependence on *z* / *L* alone. Nieuwstadt (1984) assumed a value of the von Kármán constant of 0.35 and values for a number of other closure constants that are not re-examined here. The analysis includes the change of flow direction with height. Nevertheless, the general concurrence of case 22 with the local-similarity argument given by Nieuwstadt (1984) lends support to the quality of the boundary-layer simulation.

Sorbjan (2010) presents local-similarity scaling based on four systems, necessarily in principle equivalent to each other, but some are of more practical utility as regards measurements. Here, we use his gradient-based ‘master’ scaling: a velocity scale, $U_S =\kappa zN$, and a temperature scale, $\Theta _S =\kappa z d\Theta /dz$, with an implied length scale of $\kappa {z}$, where $N^{2}=(g/\Theta )(d\Theta /dz)$. These lead to non-dimensional groups that can be expressed as functions of *Ri* alone, four of which are

$$\begin{aligned} \frac{-\overline{uw} }{U_S^2 }, \quad \frac{-\overline{w\theta } }{U_S \Theta _S }, \quad \frac{\overline{w^{2}}^{1/2}}{U_S }, \quad \frac{(\overline{\theta ^{2}} )^{1/2}}{\Theta _S }. \end{aligned}$$

**Fig. 13:**
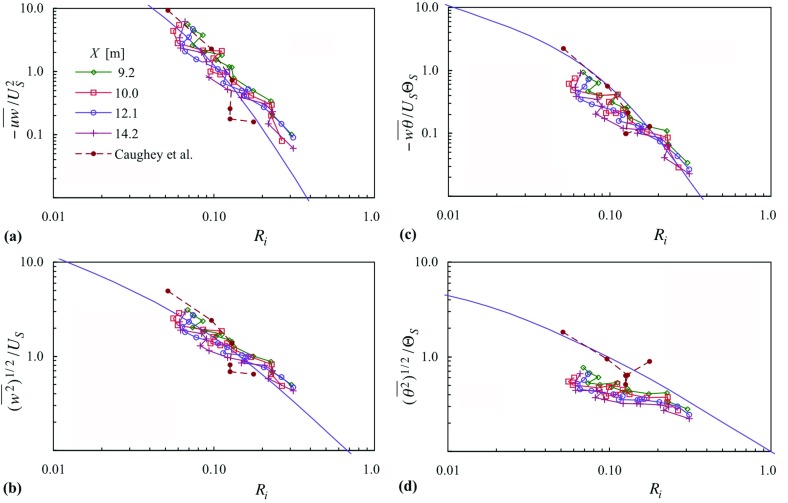
Parameters according to Sorbjan’s (2010) local scaling, for case 22. Symbols as in (**a**). Line with no symbol shows Sorbjan’s fitted curves. For Caughey et al. (1979) average values for $u_*$ and $\theta _*$: $0.16\,\hbox {m\,s}^{-1}$, 0.065 K

Sorbjan (2010) gives empirical forms for these functions based on data from the SHEBA field programme. Figure 13 gives the measurements of case 22 in terms of these scaling variables, where measurements above $z/h = 0.73$ have been excluded because of the small gradients in mean temperature and mean velocity. A similar curtailment was employed by Williams et al. (2017). Figure 13 also gives the empirical fits of Sorbjan (2010). Each of the quantities from the wind-tunnel experiments fall close to a single trend line, independent of streamwise station and consistent with horizontal homogeneity. The trends in Fig. 13a, b agree well with the empirical curves for Reynolds shear stress and $\overline{w^{2}}$, though the slope is a little less negatively steep. The trends in Fig. 13c, d are also less steep, and the general level of $(\overline{\theta ^{2}} )^{1/2}$ is about half of that given by the field data. The degree of concurrence between the wind-tunnel results and the field results seen in Fig. 13 gives a level of confidence that the simulation does indeed exhibit features of the very much higher Reynolds number of the atmosphere. It is not known why the level of the temperature fluctuation (Fig. 13d) should be below that of the field measurements while the other quantities are in much closer agreement. Figure 13 also shows the data of Caughey et al. (1979) in these scaling variables (for the same range in *z* / *h*), with very good agreement with the Sorbjan’s (2010) curves, and good agreement with the present data except for $(\overline{\theta ^{2}} )^{1/2}$. Employing the height correction of Sorbjan (2012) increases each of these quantities for both Caughey’s (1979) data and the present data (while retaining the concurrence between the different *X* stations), more so at the higher *z* / *h*. Overall, the agreement is less good.

### Obukhov Length and Surface Heat Flux

**Fig. 14:**
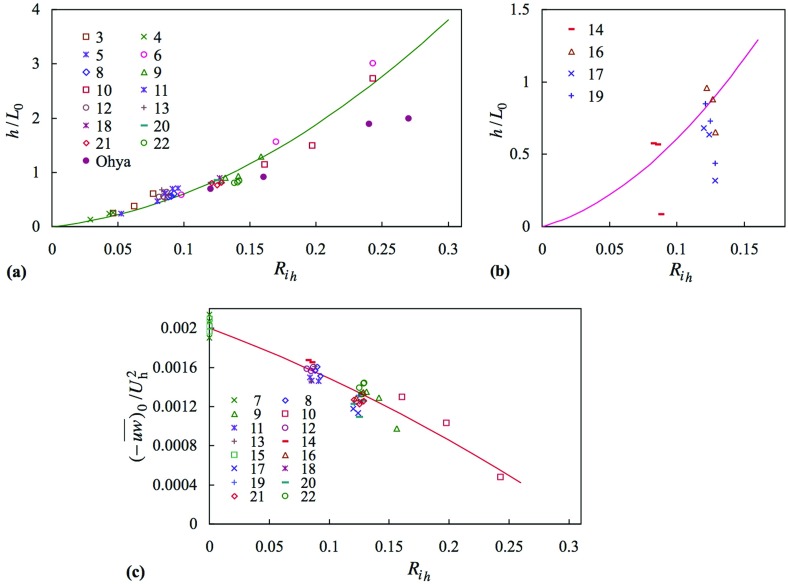
Length ratio $$h/L_0 $$ and surface shear stress as functions of bulk Richardson number. (**a**) small or negligible influence of uncooled floor length; (**b**) influence of uncooled floor length; (**c**) surface shear stress. *Legends* indicates case numbers and Ohya (2001). Line in each is given by Eq. 18 or Eq. 19

The functional relationship given by Eq. 6 assumes that the temperature of the whole of the surface is at $$\Theta _0 $$, and so will not account for an uncooled length. It also assumes a uniform temperature in the freestream. Figure 14a shows the ratio $$h/L_0 $$ for the cases where $$X_{\mathrm{C}} = 1\,\hbox {m}$$, where effectively the whole of the floor is cooled, and some other cases. These include those with and without spires and, for the latter, the case of a smooth surface. It also includes four cases where the inlet temperature profile is not uniform (cases 18, 20, 21 and 22). All the measurements fall close to a single curve, and do so for the range of Reynolds number covered (see Table 1). The measurements of Ohya (2001) exhibit a comparable but lower trend, perhaps because the surface was substantially rougher; $$z_0 /h$$ was 0.012 in neutral flow. The Reynolds number range was within that covered here. As far as the authors are aware, this form of correlation between $$h/L_0 $$ and the bulk Richardson number has not been seen before in the literature. The fitted curve (for the present data) is given by

18 $$\begin{aligned} \frac{h}{L_0 }=2.9\left( {\frac{g}{\Theta _0 }\frac{\Delta \Theta h}{U_h^{2}}} \right) +33\left( {\frac{g}{\Theta _0 }\frac{\Delta \Theta h}{U_h^{2}}} \right) ^{2}. \end{aligned}$$

That the data fall close to single curve for a variety of flow conditions gives confidence of it being of some generality. A practical utility of such a simple relationship is that for a prescribed $$h/L_0 $$ the bulk Richardson number can be straightforwardly specified; given *h* and $$U_{h}$$, $$\Delta {\Theta }$$ follows, and was used in setting up the later cases presented herein.

When there is a substantial length of uncooled floor it is to be anticipated that the heat flux would increase from zero for $$X \ge X_{\mathrm{C}}$$, but at least initially be below that implied by Eq. 18. Figure 14b shows four cases where $$X_{\mathrm{C}} \ge 5\,\hbox {m}$$. By either the second or third station $$h/L_0 $$ has risen to the level given by the curve, except perhaps for case 17. This case corresponds to the original non-uniform inlet temperature profile, where the temperature gradient near the surface is weaker than all the other cases (see Figs. 8, 9e). It is supposed that $$h/L_0 $$ for this case would continue to increase with further downstream distance to reach that given by Eq. 18. Equation 18 therefore provides an upper limit on $$h/L_0 $$.

As the temperature difference $\Delta \Theta$ increases (from zero, all else constant) the surface heat transfer will at first increase but—as the influence of stability increases, thereby reducing turbulent activity—it is to be anticipated that a maximum could be reached, with further increase in $\Delta \Theta$ leading to a *reduction* in heat transfer. This point has been demonstrated by Malhi (1995) and Mahrt et al. (1998), for example. The same behaviour can be seen in a few of the present measurements, but to see this we first need to know how stability affects the surface shear stress. The surface shear stress as a function of bulk Richardson number is shown in Fig. 14c including the neutral cases, but not the no-spire cases, which show a different behaviour. Figure  14c clearly shows a decreasing trend over and above the scatter, indicative of the reduction in turbulence levels seen in earlier figures. For the trend line shown

19 $$\begin{aligned} \frac{u_{*}^{2}}{U_h^{2}}=0.0020 \left[ {1-2.3 \left( {\frac{g}{\Theta _0 }\frac{\Delta \Theta h}{U_h^{2}}} \right) -3.0 \left( {\frac{g}{\Theta _0 }\frac{\Delta \Theta h}{U_h^{2}}} \right) ^{2}} \right] . \end{aligned}$$

The constant term on the right-hand side of this equation pertains to the state of the neutral boundary layer, which would be different in different circumstances, and so this equation cannot be regarded as general.

By writing the right-hand sides of Eqs. 18 and 19 as $Q(Ri_{\mathrm{h}} )$ and $P(Ri_{\mathrm{h}} )$, respectively, and the surface Obukhov length in terms of surface heat flux, Eq. 18 can be written as

20 $$\begin{aligned} -(\overline{w\theta } )_0 \frac{g}{\Theta _0 }\frac{h}{U_h^{3}}=\frac{1}{\kappa Ri_{\mathrm{h}} }\;[P(Ri_{\mathrm{h}} )]^{3/2}Q(Ri_{\mathrm{h}} ). \end{aligned}$$

**Fig. 15:**
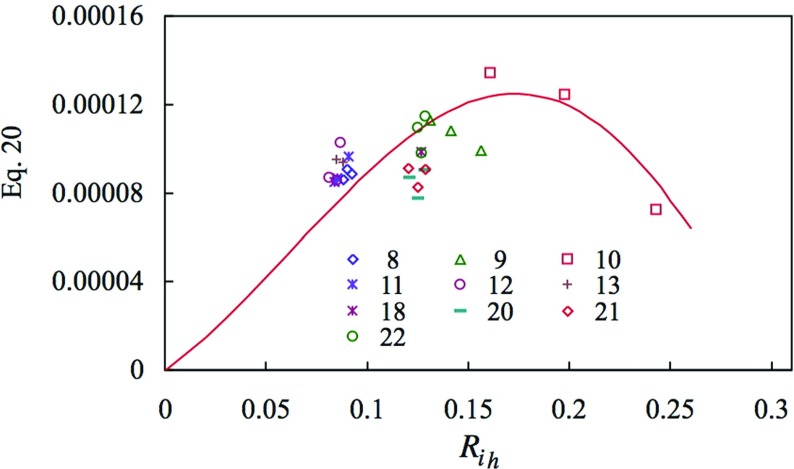
Non-dimensional surface heat flux of Eq. 20, as a function of bulk Richardson number. *Symbols*: measured quantities. *Line*: from fitted lines of Fig. 14

Figure 15 shows the left-hand side of Eq. 20 as given by the measurements, and the right-hand side as given by the fitted curves. The behaviour of the surface heat flux as a function of bulk Richardson number is such that it exhibits a very clear maximum, though the sharp decrease to the right of the maximum is based on only a few points. Nevertheless, the shape is comparable with that given by Malhi (1995) and Mahrt et al. (1998). In this figure the maximum arises at about ${ Ri}_{\mathrm{h}} = 0.17$ which, from Fig. 13, corresponds to $h/L_0 = 1.5$. For the maximum,

21 $$\begin{aligned} (\overline{w\theta } )_0 \frac{g}{\Theta _0 }\frac{h}{U_h^{3}}\approx 0.00013. \end{aligned}$$

Mahrt et al. (1998) define the boundary-layer state to the left of the maximum heat transfer as weakly stable, and to the right as transitionally stable, and further to the right as very stable. In contrast to Malhi (1995) and Mahrt et al. (1998), the maximum here is given in terms of $$h/L_0 $$ rather than $$z/L_0 $$, where in the latter paper the authors note that there are different values of $$z/L_0 $$ for the maximum from different datasets. It is tentatively suggested that the present result, based on $$h/L_0 $$, might be of more generality. Case 10 in Fig. 6 is one in which the $${ Ri}_{\mathrm{h}}$$ exceeds 0.18 with marked changes in mean velocity profiles and marked reductions in Reynolds stresses, and might be described as transitionally stable. In that, for case 22, $${ Ri}_{{h}} = 0.14$$ is fairly close to that for maximum heat transfer, where stability effects have become strong enough to halt further increase, we describe this case as moderately stable.

## Further and Concluding Comments

The results show that it is possible to generate a thick, moderately stable boundary layer that is approximately horizontally homogenous and follows quite closely local-scaling behaviour as proposed in Nieuwstadt (1984) and Sorbjan (2010), with support also from the full-scale data of Caughey et al. (1979). In the experimental set-up used here, the boundary-layer height is determined by the ‘spire-type’ flow generators. For weak or moderate stability the flow generators essentially set the shape of the mean velocity profile, and only the profile shapes of the Reynolds stresses and other turbulence quantities are affected by stability. A uniform inlet temperature does not give a boundary layer that is stable across its full depth, but results in the upper approximately 1/3 remaining neutral. A non-uniform inlet profile is essential in order to achieve stability over the full depth, but needs to be carefully specified. The type of profile used here is not unique, and it is assumed that further refinement would allow greater control of, for example, the profile of Richardson number across the layer.

The profiles of Reynolds stresses and other quantities are also affected by the distance from the inlet at which surface cooling is started. Over an initially uncooled length, the flow develops from that created by the flow generators and the inlet temperature profile. If the profile is uniform this will, of course, be the development of an initially neutral flow, while for a non-uniform profile the effects of stability are also developing, but without the large temperature gradient that arises once the flow has moved beyond the start of the cooled surface. The cooling must lead to the growth of an internal layer; the results show that if it is started too soon the profiles of the Reynolds stresses and other quantities have shapes that do not change monotonically over the depth of the layer. Such a layer is of course a stable boundary layer, but the strategy here (at this stage) has been to seek profiles, such as those of Reynolds stresses, turbulent heat flux and temperature fluctuation that, as with the measurements of Caughey et al. (1979), decrease smoothly with increasing height.

Although there is no direct correspondence, it is interesting to note that Mason and Derbyshire (1990) started their large-eddy simulation in a neutral state before applying surface cooling (after unsatisfactorily starting from a stratified initial condition), rather like the simple observation made in Sect. 2 for a stable layer growing spatially from zero thickness. Here, though, constraining the initial development of the layer to be neutral does not allow a satisfactory development.

The finding that the mean velocity profile is little affected for weak or moderate stability suggests that a specific mean velocity profile could be prescribed by appropriately-shaped flow generators. Control of the inlet temperature profile shape and the point at which surface cooling is started would then be used to control the profiles of the various turbulence quantities. The closeness of the mean velocity and mean temperature profiles of case 22 with those given by Caughey et al. (1979) was not expected, though it is certainly pleasing to see. A relatively small modification to the flow generators should allow a closer match of mean velocity, should it be desired, and fine tuning of the inlet temperature profile may allow a closer match of the downstream profile. (For a facility such as that used here it may require control of the inlet heaters near the surface that is finer than the current heater-bank unit height of 100 mm.)

The care with which the inlet temperature profile has to be specified is a consequence of the sensitivity of the developed flow to initial conditions. That is, the particular shape of an inlet temperature profile has a long-lasting influence. Given that the initial conditions of a stable boundary layer in the real atmosphere are the conditions ‘inherited’ from the daytime boundary layer, and the variability seen in the night-time boundary layer (e.g. Hunt 1985), the behaviour seen in this investigation is not surprising. The stable layer is fundamentally different from that of a neutral layer.

The closeness of the correlation represented by Eq. 18 and Fig. 14a cannot be general, as is demonstrated by the argument leading to this equation, and from Fig. 14b. There is of course an aspect of self-correlation, in that the height *h* appears on both sides, though the relationship is non-linear and *h* is in any case fixed by the height of the flow generators. It appears to apply sufficiently far downstream independently of the length of uncooled surface, surface roughness or inlet temperature profile shape. Clearly, it would be of interest to test its practical utility more widely, addressing such questions as its behaviour with higher surface roughness or the imposition of an overlying inversion.

As mentioned earlier, the behaviour represented by Eq. 19 is more restricted. *If* the part inside the square brackets on the right-hand side of Eq. 19 is of more generality than the equation itself, then the maximum of the curve in Fig. 15 would occur at the same bulk Richardson number $g \Delta \Theta h/\Theta _0 U_h^{2}$ of about 0.17. Here, the term moderately stable is used to denote a boundary layer in which the bulk Richardson number is comparable with but less than that for maximum surface heat transfer. The two points beyond the maximum are for case 10, where the mean velocity profile shape has started to change significantly (Fig. 6), and might be described as transitionally stable. In the absence of a significant inversion a value for the bulk Richardson number might be a more general means of defining the condition for maximum surface heat flux, rather than the *z*-dependent approach of Malhi (1995) and Mahrt et al. (1998).

The strategy adopted in achieving the results presented (in Sects. 4.4–4.6) is expected to apply to rougher surfaces (already partly done by Marucci et al. 2016), and to higher Reynolds number flow. As the present roughness was low, being nominally representative of a sea surface, a rougher surface is easily achieved, but the Reynolds number is in practice heavily constrained, principally by the power and energy required to achieve the desired stratification. (The power required varies as the cube of the freestream velocity, and the temperature difference as the square, for a given level of stability.) The results in Figs. 14 and 15 suggest that the influence of Reynolds number is weak; the dominant influence is that of Richardson number. The concurrence seen in Figs. 12 and 13 also indicates no more than a weak influence of Reynolds number.

## Footnotes

1 Ignoring intermittency.

2 Case 15 is a repeat of case 7, and is retained for the same reason.

3 The roughness elements and the density of the elements were identical; the layout was different, the pattern here repeating at rows 5, 9, 13.. rather than 3, 5, 7.. . This revised pattern was regarded as ‘more random’.
